# Radical-induced single-molecule conductance tuning in 9,9′-bifluorenylidene derivatives†

**DOI:** 10.1039/d4sc07256a

**Published:** 2025-02-10

**Authors:** Hanjun Zhang, Lichuan Chen, Yunzhu Huang, Xiaodong Liu, Sergio Moles Quintero, Wenjing Hong, Dongsheng Wang, Juan Casado, Yonghao Zheng

**Affiliations:** a School of Optoelectronic Science and Engineering, University of Electronic Science and Technology of China (UESTC) Chengdu 611731 People's Republic of China zhengyonghao@uestc.edu.cn xdliu@uestc.edu.cn wangds@uestc.edu.cn; b State Key Laboratory of Physical Chemistry of Solid Surfaces, College of Chemistry and Chemical Engineering Xiamen University Xiamen Xiamen 361005 People's Republic of China; c Department of Physical Chemistry, University of Málaga Campus de Teatinos s/n Málaga 29071 Spain; d State Key Laboratory of Organic Electronics and Information Displays & Institute of Advanced Materials (IAM), Nanjing University of Posts & Telecommunications 9 Wenyuan Road Nanjing 210023 People's Republic of China

## Abstract

Single-molecule techniques provide new perspectives for understanding the relationship between spin delocalization of organic radicals and the intramolecular electronic structure. In this study, a series of 9,9′-bifluorenylidene (9,9′-BF) derivatives with four functionalization sites were synthesized, showcasing the orthogonalization of non-conducting (between radical sites) and conducting (between thiomethyl groups) paths. By precisely controlling the amount of radicals, or radical injection, ranging from mono-radical (Mono-PFPR) to diradicals, and among these, varying from medium (Di-PFPR*y*_0_ = 0.66) to small (Di-PFNR*y*_0_ = 0.11) to vanishing (Di-NFNR*y*_0_ = 0) diradical characters (*y*_0_ represents the diradical index), the influence of organic radical spin delocalization on the conducting path can be gradually modulated, transforming linear conjugated conducting channels into cross-conjugated channels and significantly reducing single-molecule conductance. This discovery provides an in-depth understanding of the complex relationship between radicals, spin delocalization, and molecular conductance, which is rather unique in the area of functional stable radical compounds. Ultimately, it provides forward-looking guidance for research on these materials in the field of organic electronic materials.

## Abstract 2

A series of 9,9′-bifluorenylidene derivatives demonstrate orthogonalization of non-conducting (between radical sites) and conducting (between thiomethyl groups) paths. Precise radical injections significantly reduce single-molecule conductance.

## Graphical abstract

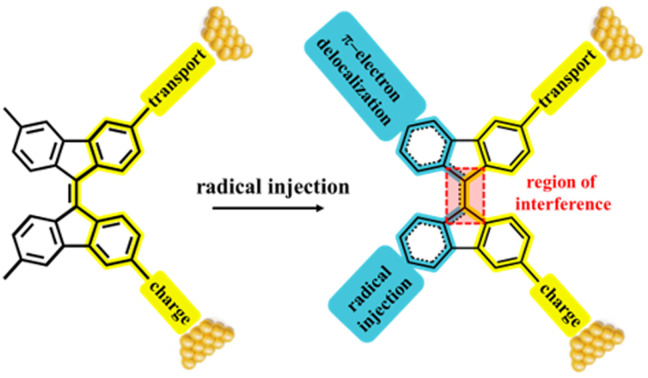

## Introduction

Molecular electronics is an emerging field that studies charge transport through molecules, encompassing knowledge from chemistry, materials science, physics, and micro/nanofabrication.^1–6^ Among these, single-molecule techniques, applied to the case of open-shell radical and diradical molecules, can offer unique advantages in investigating spin properties and transport mechanisms to provide a comprehensive understanding of the intrinsic characteristics of these organic radicals.^7–23^ However, there are still significant gaps in the establishment of the mechanisms relating electrical transport to spin characteristics at the molecular scale. In particular, the impact of intramolecular spin delocalization properties on the electrical conductance remains largely unexplored.

Recent advancements in single-molecule techniques have provided unprecedented insights into the behaviour of organic radicals at the single-molecule level.^24–27^ Different monoradicals (*e.g.*, Blatter,^8–10^ PTM^11^ or TEMPO^12–14^-containing radicals) have been studied at the single molecule level. Recently, our group achieved bidirectional conductance modulation in single-molecule junctions by using electronic injection from off-site neutral radicals acting as gating terminals.^20^ Van der Zant *et al.* discovered that the single-molecule junction with the perchlorotriphenylmethyl (PTM) radical exhibited the Kondo effect which is unaffected by the mechanical and electrostatic changes in both two- and three-terminal solid-state devices.^11^ The phenomenon of high positive magnetoresistances (MRs) observed by Scheer *et al.*, ranging from 16–287% under a 4 T magnetic field, in comparison to the 2–4% observed in non-radical ligo(*p*-phenyleneethynylene) (OPE) molecules, could potentially be explained by the disruptive effect of the applied magnetic field coupled with the spin momentum.^14^

Alternatively, Hong,^7^ Vezzoli,^8^ and our group^23^ proved that introducing radicals into molecular systems is also a good choice for enhancing/modulating conductance. Single-molecule devices have the unique advantage of studying the spin properties and intrinsic electrical characteristics of single/isolated organic radical molecules.

**Scheme 1:**
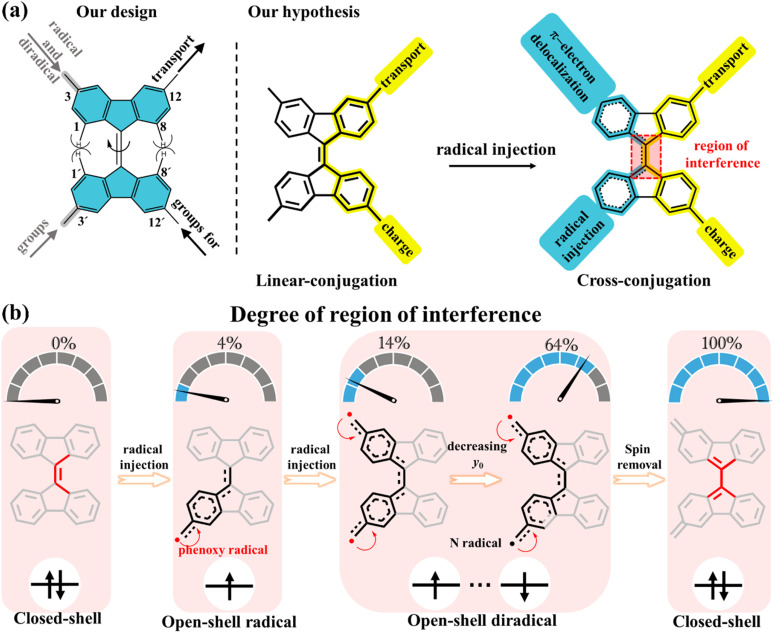
Schematic of 9,9′-bifluorenylidene (blue shaded) derivatives. (a) Our design of modulation of electronic properties and processes by orthogonal functionalization through the 12,12′-positions (conductance channel, highlighted in yellow) and the 3,3′-positions (injection/delocalization channel, highlighted in blue). Highlighted in red is the molecular segment where interference takes place. (b) Schematic of the degree of region of interference for radical injection. The percentages 0%, 4%, 14%, 64%, and 100% represent the influence of spin delocalization on the core structure of 9,9′-BF, here, 0% indicates that the 9,9′-BF core structure in the non-radical compound is unaffected by spin, while 100% signifies that the diradical formation leads to a closed-shell structure, thoroughly altering the 9,9′-BF core structure. The numbers 4%, 14%, and 64% are derived from theoretical calculations corresponding to the spin population highlighted in red boxes within Fig. 3a.

In our present strategy, 9,9′-bifluorenylidene (9,9′-BF) serves as a core structure to investigate the impact of intramolecular spin delocalization on electrical transport properties, as schematically depicted in Scheme 1a. This structure features multiple functionalization sites and enables modulation of electronic properties and processes through orthogonal functionalization at the 12,12′-positions (conductance channel, highlighted in yellow) and the 3,3′-positions (injection/delocalization channel, highlighted in blue). Additionally, tuned radical injection from lateral groups over the main conductance channel allows for precise control of the quantity of radical injection while maintaining the integrity of the charge transfer pathway. 9,9′-BF is made of overcrowded alkenes composed of two planar π-conjugated fluorenes in which the steric repulsion by the proximity of the hydrogen atoms at the 1,8- and 1′,8′-positions drives a potential twist around the alkene.^28–30^ The degree of C=C twisting partially weakens the double bond strength, a C=C character that is additionally modulated by the degree of spin delocalization/injection between the 3,3′-positions to the extent that the 12,12′-conducting path eventually changes from linear conjugation to cross conjugation after radical injection (Scheme 1a). In other words, a lack of spin delocalization in the monoradicals or non-interspin delocalization in the diradicals produces a 3,3′path with enlarged aromatic structures in their benzenoid rings. This implies that vanishing interference with the conducting path occurs, where linear conjugation dominates. Conversely, increased spin delocalization over the 3,3′path induces partial quinoidization of the rings forcing the coupling with the 12,12′path through cross-conjugation.

Radicals are known to induce cleavage and reorganization of multiple chemical bonds within molecules due to the resonance or delocalization of the unpaired electron.^31–33^ This process can lead to molecular twisting, deformation, or molecular reshaping. In this study, we explore the impact of radical injection on 9,9′-BF conductance by adjusting substituents at the 3,3′-position. Initially, we control the number of free electrons—mono-radical and diradical—as illustrated in Scheme 1b. Diradicals generally exhibit a greater influence on the central 9,9′-BF skeleton compared to mono-radicals due to their spin–spin coupling properties. The extent of diradical character is quantified by the diradical index (*y*_0_),^31^ ranging from *y*_0_ = 0 for closed-shell molecules to *y*_0_ = 1 for fully open-shell species, with 0 < *y*_0_ < 1 indicating partial olefin double bond rupture. As shown in Scheme 1b, the lower the *y*_0_ value, the greater the π bond rupture.

Next, by careful design of the chemical structure, the enhanced spin–spin coupling strength between the two unpaired electrons reduces *y*_0_ and enhances their impact on the 9,9′-BF skeletons. Ultimately, by minimizing the distance between the radicals, the total spin density is removed by which we successfully achieve a quinone-like structure that transforms the main conductance pathway from linear to cross conjugation. The novelty in our research lies in aggregating the electronic impacts of incorporating radical and diradical segments with the understanding of their interplay through single-molecule conductance.

**Scheme 2:**
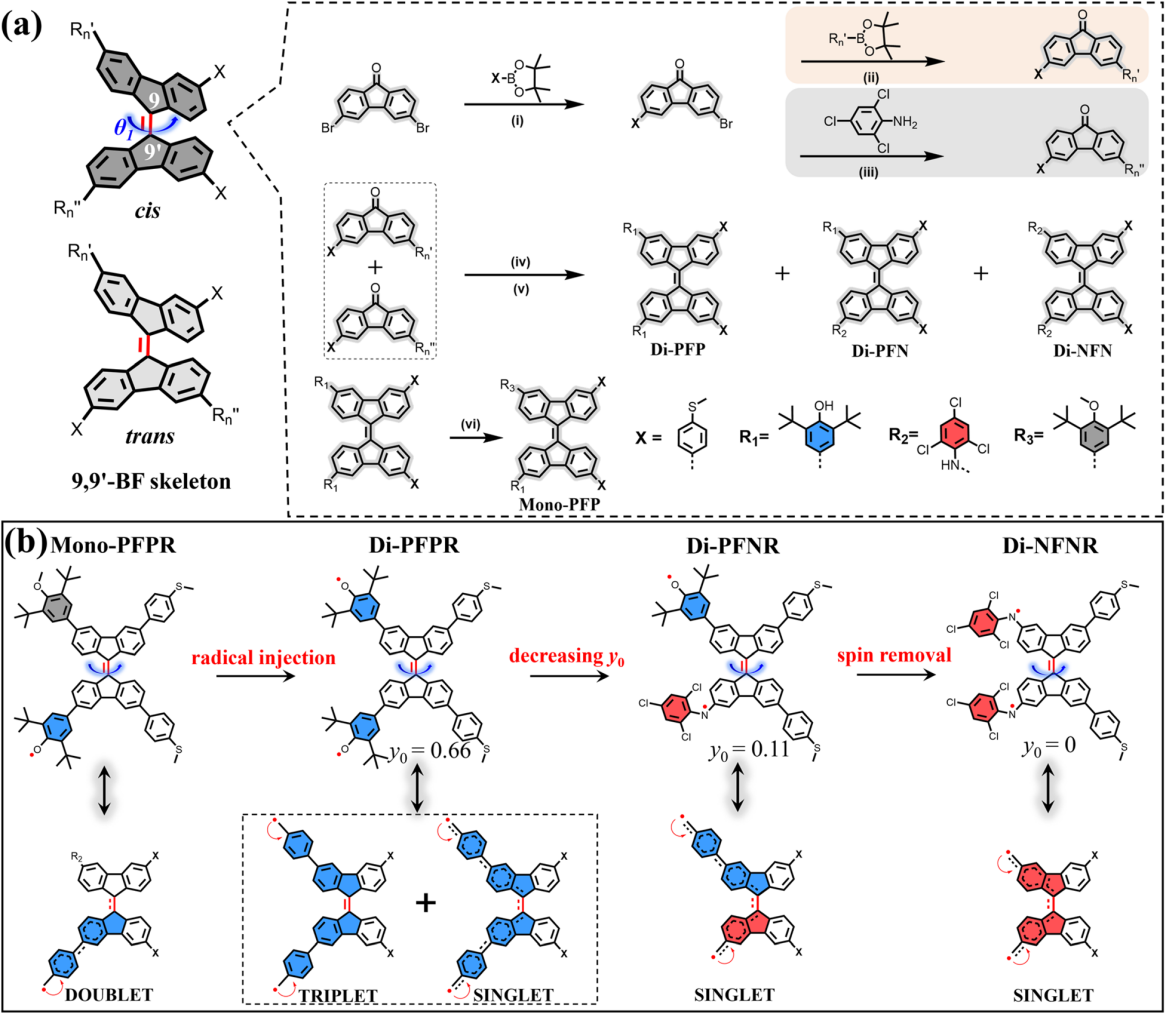
(a) Synthetic routes of non-radical compounds. Reagents and conditions: (i) Pd(PPh_3_)_4_, SMe-pinacol boronic ester, KOH, H_2_O, toluene, 90 °C; (ii) Pd(PPh_3_)_4_, relative reactant pinacol boronic ester, KOH, H_2_O, toluene, 90 °C; (iii) Pd_2_(dba)_3_, BINAP, sodium *tert*-butoxide, toluene, 120 °C; (iv) Lawesson's reagent, toluene, 120 °C; (v) copper powder; (vi) CH_3_I, *N*,*N*-dimethylformamide, rt.; (b) resonance canonical forms of all *cis* radicals. The *trans* isomers are not discussed due to undetectable conductance in STM-BJ.

We have designed and synthesized a series of radical based molecules with a 9,9′-BF molecular skeleton and methyl sulfide (SMe) anchoring groups, as illustrated in Scheme 2a. According to the different substituents (
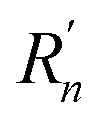
 and 
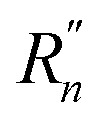
 at the 3,3′-positions), these compounds, collectively referred to as 9,9′-BF derivatives, are named Mono-PFP, Di-PFP, Di-PFN and Di-NFN. The synthetic routes for all molecules are depicted in Scheme 2a, with detailed descriptions of the synthetic process available in the ESI (Fig. S1).† Briefly, the mono-radical (Mono-PFPR), diradicals (Di-PFPR and Di-PFNR) and the quinone-like compound (Di-NFNR) can be produced by the oxidation of the corresponding non-radical molecules with lead dioxide. Furthermore, we controlled the interaction between unpaired electrons in these molecules by altering the chemical structure as shown in Scheme 2b. By replacing one of the phenoxyl radicals in Di-PFPR with an N-radical, we obtained Di-PFNR which exhibits stronger interaction between unpaired electrons than Di-PFPR and thus a smaller *y*_0_ value. Moreover, by replacing the remaining phenoxyl radical in Di-PFNR with an N-radical, we obtained Di-NFNR which turns out to bear a quinone-like structure. As shown in Scheme 2b, it is worth noting that non-radical compounds (Mono-PFP, Di-PFP, Di-PFN and Di-NFN) exhibit *cis* and *trans* stereoisomeric structures. We attempted to obtain the *trans*-9,9′-BF structure using the photoisomerization method, as illustrated in Fig. S2,† with the molecule Mono-PFP as an example. The absorption spectrum of the photo-responsive moiety in Mono-PFP spans from 250 to 650 nm. However, after irradiation with 365, 470, and 590 nm LED lamps (20 mW cm^−2^) for 120 minutes, almost no changes were observed in the absorption spectrum, indicating that light cannot induce the *trans*-to-*cis* isomerization. Similar to the phenomenon observed in the absorption spectrum, the ^1^H NMR spectrum of Mono-PFP shows almost no change after irradiation with 365, 470, and 590 nm LED lamps (20 mW cm^−2^) for 120 minutes, which further verifies that light cannot induce the *trans*-to-*cis* isomerization (Fig. S3†). In addition, we used molecule Mono-PFP as an example; thus, the ^1^H nuclear magnetic resonance (NMR) spectroscopy confirms the presence of stereoisomers (*cis* and *trans*) by observing the splitting patterns of peaks in the range of 7 to 9 ppm (Fig. S4†). Additionally, two-dimensional ^1^H NMR indicates the presence of the *trans* configuration in Mono-PFP (Fig. S5†) (the detailed analysis can be found in the ESI†). However, in this study, we only focused on the *cis* structure given that it is the only one that showed conductance in the single-molecule measurements. Specifically, the theoretically calculated molecular lengths of the *cis* and *trans* isomers are 1.7 nm and 2.1 nm (Fig. S10†), respectively. The lengths of all molecular junctions are about 1.7 nm determined from the relative-displacement distributions of the 9,9′-BF derivatives (Fig. S9†), which correspond to the *cis* structure. Our molecular strategy, in Scheme 2b, considers the constant steric strain in the 9,9′-BF unit, as imposed by the H–H repulsion, whereas the structural diversification is introduced in the cross-conjugated 3,3′and 12′,12-positions of the 9,9′-BF unit. The following points highlight the relevance of the designs:

(i) In the 3,3′-positions, radical (Mono-PFPR) and diradical species with different diradical characters (Di-NFNR with *y*_0_ = 0 and full π-electron delocalization, Di-PFNR with *y*_0_ = 0.11 and medium π-electron delocalization and Di-PFPR with *y*_0_ = 0.66 and diminished π-electron delocalization) lead to different degrees of inter-fluorene π-electron delocalization at the same time that represent a modulation of the amount of radical injection, going from radical to diradicals.

(ii) In the 12′,12-positions, electroactive SMe groups that form a channel for molecular conductance are incorporated which are able to link the two extremities to gold tips for STM-BJ measurements.

## Results and discussion

**Fig. 1:**
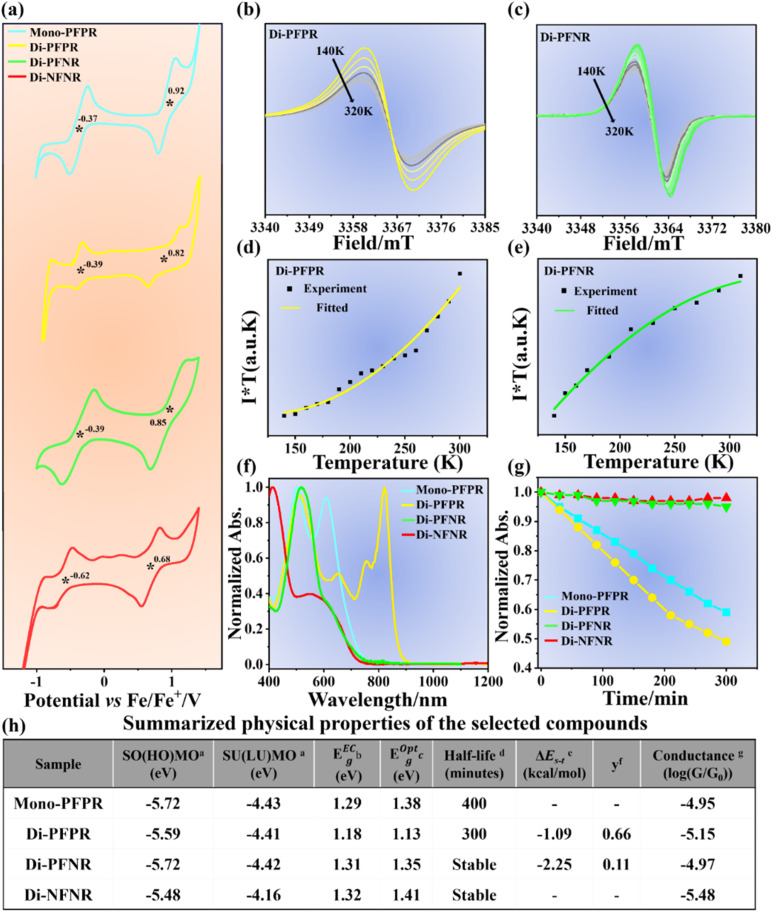
(a) Cyclic voltammograms of Mono-PFPR, Di-PFPR, Di-PFNR and Di-NFNR in dry DCM with 0.1 M nBu_4_NPF_6_ as the supporting electrolyte, Ag/AgCl as the reference electrode, glassy carbon as the working electrode, Pt wire as the counter electrode, and a scan rate of 50 mV s^−1^. The *VT* ESR spectra of (b) Di-PFPR and (c) Di-PFNR from 140 to 310 K in benzophenone solid solution. The measured (black square) and fitted (solid line) *I* × *T*–*T* curves of (d) Di-PFPR and (e) Di-PFNR based on the *VT* ESR measurements. The solid lines are the fitting curves according to the Bleaney–Bowers equation. (f) Normalized absorption spectra of Mono-PFPR, Di-PFPR, Di-PFNR and Di-NFNR in DCM at a concentration of 10^−4^ M in air, and (g) absorbance decay at the maximum absorption peak and fitting curve, which can be fitted with equation *I*(*t*) = *I*_0_(1/2)^(*t*/*τ*)^ to determine the half-life (*τ*); (h) the summarized physical properties of the selected compounds. ^a^SO(HO)MO and SU(LU)MO were calculated from cyclic voltammetry; ^b^E^Ec^_g_: electrochemical SO(HO)MO–SU(LU)MO energy gap; ^c^E^opt^_g_: optical energy gap estimated from the onset of the absorption spectra; ^d^the chemical stability of the radicals by monitoring the time evolution of their UV-vis-NIR absorption spectra under ambient conditions; ^e^Δ*E*_S–T_ was estimated by fitting the *I* × *T*–*T* curves with the Bleaney–Bowers equation; ^f^calculated at the level of UM062x/6-311G(d) based on the occupation number of the lowest unoccupied natural orbital (LUNO). ^g^Single-molecule conductance measured *via* STM-BJ.

We studied the electrochemical properties of Mono-PFPR, Di-PFPR, Di-PFNR and Di-NFNR using cyclic voltammetry (CV) in dry dichloromethane (DCM). Fig. 1a shows oxidation/reduction peaks for all compounds which are expected for molecules with an electron richer SMe path, where oxidation would take place, and with an electron deficient path constituted by the open-shell and quinoidal groups where the reduction processes would likely occur.

The formation of radicals was verified by electron spin resonance (ESR) and UV-vis-NIR absorption spectroscopy. The ESR measurements were carried out in anhydrous toluene at 295 K. All the radical samples show distinct ESR signals with *g* values of 2.003, which unambiguously confirm their existence in solution (Fig. S6†). In diradicals, the presence of two unpaired electrons with specific spin and orbital characteristics leads to the observation of multiple *g*-factor components, including the *g*4 component. This *g*4 signal serves as a specific marker for diradicals and is not observed in the spectra of the mono-radical (Fig. S7†).

The magnetic properties of Di-PFPR and Di-PFNR diradicals were studied by the variable-temperature (*VT*) ESR measurements from 140 to 310 K in benzophenone solid solution (Fig. 1b and c). The intensity (*I* × *T*) of double integral ESR intensity (*I*) multiplied by temperature (*T*, in K) decreases with the lowering of temperature, which indicates that all diradicals have an open shell singlet ground state (S_0_). The singlet–triplet energy gap (Δ*E*_S–T_) is roughly estimated by fitting the *I* × *T vs. T* curve with the Bleaney–Bowers equation,^34^ yielding the Δ*E*_S–T_ values of −1.09 and −2.25 kcal mol^−1^ for Di-PFPR and Di-PFNR, respectively (Fig. 1d, e and h). The calculated Δ*E*_S–T_ values are −0.75, and −1.73 kcal mol^−1^ for Di-PFPR and Di-PFNR, respectively, consistent with the experimental results obtained from the *I* × *T vs. T* curves. Di-PFNR exhibits the larger Δ*E*_S–T_ value, indicating stronger interaction between the radicals within the molecule, consistent with its smaller diradical character, *y*_0_ = 0.11. Meanwhile, the smaller Δ*E*_S–T_ and larger *y*_0_ = 0.66 of Di-PFPR than Di-PFNR are consequences of its larger number of Clar sextets recovered in the diradical state. However, a comparative evaluation of these properties between the two systems Di-PFPR and Di-PFNR must be done cautiously due to the fundamental distinctive features of symmetric and asymmetric diradicals, respectively. Note that no ESR signal is observed for Di-NFNR, implying its closed-shell (*y*_0_ = 0) quinone-like structure (Fig. S6†).

UV-vis-NIR absorption spectra of these open-shell molecules in Fig. 1f show broad absorption bands in the wavelength range of 600–900 nm. Furthermore, we examined their chemical stability by monitoring the change in the absorption peak intensity as a function of time under ambient conditions. The absorption intensities of Mono-PFPR and Di-PFPR, gradually decrease over time, with an estimated half-life (*t*_1/2_) of 400 and 300 min, respectively (Fig. 1g and h). It is noted that the absorption peak intensity of Di-PFNR remains unchanged over time (Fig. S8†), similar to that observed in the closed-shell quinone-like Di-NFNR.

**Fig. 2:**
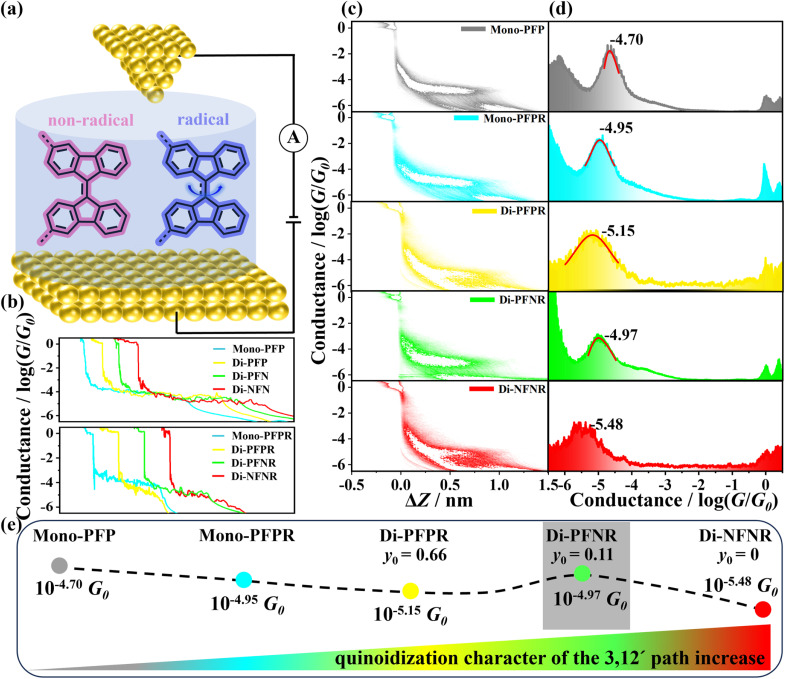
(a) The schematic STM-BJ measurements in TCB solution; (b) the typical individual conductance traces of Mono-PFP, Di-PFP, Di-PFN, Di-NFN, Mono-PFPR, Di-PFPR, Di-PFNR and Di-NFNR; (c) the 2D conductance–displacement histograms of Mono-PFP, Mono-PFPR, Di-PFPR, Di-PFNR and Di-NFNR; (d) 1D conductance histograms of Mono-PFP, Mono-PFPR, Di-PFPR, Di-PFNR and Di-NFNR; (e) the schematic diagram illustrating the variation trend of conductance with the quinoidal character.

Single-molecule conductance measurements of all the compounds in degassed 1,2,4-trichlorobenzene (TCB) solutions were carried out using the STM-BJ technique at room temperature in air (Fig. 2a). For all radicals, these experiments were performed within 10 min after purification. The measuring time was in the range of several tens of minutes to half an hour.

Fig. 2b shows the individual conductance–displacement traces of all compounds. Each trace exhibits a distinct plateau at *G*_0_ (quantized conductance, *G*_0_ = 2*e*^2^/*h*) associated with the gold contact, and an additional plateau at a molecule-dependent conductance value below *G*_0_, due to conductance through a molecule bonded in the gap between the two gold point contacts.^35^ The individual traces of mono- and diradical compounds show more slopy profiles compared to those of non-radical compounds, which suggests that the mono- and diradicals experience more significant conformational changes during their stretching on the molecular junctions. This is primarily due to the injection of radicals, which gradually disrupt the central double bonds of the 9,9′-BF moieties, resulting in molecular deformation upon stretching. Considering that 9,9′-BF derivatives in TCB solution may exist as *cis* and/or *trans* isomers, we identify the predominant one by means of the STM-BJ technique. The theoretically calculated molecular lengths of the *cis* and *trans* isomers are 1.7 nm and 2.1 nm (Fig. S10†), respectively. From the relative-displacement distributions of the 9,9-BF derivatives (Fig. S9†), the lengths of the molecular junctions are determined to be approximately 1.7 nm after adding the gold–gold snap-back distance of ∼0.5 nm,^36^ in good agreement with the theoretically calculated molecular length of 1.7 nm of the *cis* isomer. As shown in Fig. S11,† we calculated the transmission spectrum of the *trans* isomer of Mono-PFP and found that its transmission coefficient *T*(*E*) is significantly lower than that of the *cis* isomer around the Fermi energy (*E*_F_) (*i.e.*, *E* − *E*_F_ ≈ 0 eV) due to the presence of destructive interference. Therefore, we speculate that the conductance of the *trans* isomer may be below the detection limit of the instrument, resulting in its conductance not being detected. Furthermore, we attempted to detect the conductance of the *trans* isomer by increasing the bias voltage, as shown in Fig. S12.† However, even with the increased bias voltage, only a single conductance peak was observed.

Fig. S9† presents the one-dimensional (1D) conductance histograms for the corresponding non-radical compounds which were fitted to Gaussian functions resulting in the most probable conductance values of these non-radical molecules, Mono-PFP, Di-PFP, Di-PFN, and Di-NFN which are 10^−4.70^*G*_0_, 10^−4.72^*G*_0_, 10^−4.76^*G*_0_ and 10^−4.72^*G*_0_, respectively. Their similar conductance values suggest that the substituents in 9,9′-BF derivatives scarcely affect the total molecular conductance.

Fig. 2c shows the two-dimensional (2D) conductance–displacement histograms, constructed from over 2000 independent traces, for Mono-PFP, Mono-PFPR, Di-PFPR, Di-PFNR, and Di-NFNR. All molecules display well-defined conductance clouds, indicative of the formation of Au-molecule-Au junctions. Further analysis of the relative-displacement distributions of the mono- and diradicals shows that the lengths of all molecular junctions are approximately 1.7 nm (Fig. S15†), similar to that of the non-radicals (Fig. 2a). Fig. 2d presents the 1D conductance histograms of Mono-PFP, Mono-PFPR, Di-PFPR, Di-PFNR, and Di-NFNR, showing neat conductance peaks at 10^−4.70^, 10^−4.95^, 10^−5.15^, 10^−4.97^, and 10^−5.48^*G*_0_, respectively. Compared to the conductance of the non-radical Mono-PFP (10^−4.70^*G*_0_), the introduction of one radical into Mono-PFPR gives rise to a remarkable decrease in the conductance (10^−4.95^*G*_0_). In the non-radical Mono-PFP, the central olefin has double bond character by which the inclusion of the radical in the non-conducting channel induces the partial weakening of the central olefin double bond, which imparts a decrease in the conductance.

There are recent reports of molecules binding to gold through deprotonated oxygen and imines both of which are known to bind robustly to gold electrodes. Therefore, we designed and synthesized diradicals 4FR and 4NR as reference compounds without SMe anchoring groups. Their 1D and 2D conductance histograms do not show any detectable conductance features (Fig. S16†), which rules out the possibility that deprotonated oxygen or imines might act as anchoring groups.

On passing from Mono-PFPR to Di-PFPR, π-electron delocalization between the two semiquinone groups in the singlet state induces the partial quinoidization of the 3,3′path, which would further interfere with the 12,12′conductive channel imparting a reduction of the conductance from Mono-PFPR to Di-PFPR. In Di-PFPR, the large diradical character further produces a high spin triplet state (see the above section) very close in energy and to some extent thermally populated at 298 K. In that triplet state, a full aromatic character would be recovered in the 3,3′path mitigating the interference effect existing in the singlet state. Therefore, going from mono(radical) to bis(radical) triplet, an increase in conductance is expected (*i.e.*, the interference effect can be conveniently corrected/attenuated/diminished by the partial thermal population of the triplet state originating from the singlet ground electronic state). Further strengthening of the quinoidal path in Di-NFNR by reduction of the number of benzene rings accentuates the interference between the two paths in the conduction state, hence imparting the maximal reduction of the measured conductance.

Di-PFNR is an asymmetric diradical which contrasts with the symmetric diradicals Di-PFPR and Di-NFNR. The asymmetry between the energy sites of the different unpaired electrons established an energy gap (non-existing in symmetric diradicals) or a push–pull effect by donor–acceptor coupling that reduces the diradical character and favors a closed-shell zwitterionic state. Such a zwitterion would recover the aromatic character in the imine and semiquinone moieties, which would diminish the interference with the conducting channel and consequently increase the conductance. This can explain why Di-PFNR exhibits higher conductance than Di-PFPR and Di-NFNR.

Flicker noise measurements on the conductance plateaus were also carried out. We suspended the tip for 150 ms once a conductance plateau occurred (see the ESI† for details) and collected thousands of conductance–time traces. Then, we analyzed the flicker noise power spectral density (PSD) against the corresponding average junction conductance (*G*). Given that the PSD of the junction with through-bond coupling transmission scales as *G*^1.0^, its normalized PSD (PSD/*G*) shows the non-correlation to *G*.^37^ Fig. S17† presents the 2D histograms of the normalized PSD *versus G*. The noise powers of Mono-PFP, Di-PFP, Di-PFN, and Di-NFN scale as *G*^1.1^, *G*^1.2^, *G*^1.1^, and *G*^1.2^, respectively, indicating the through-bond dominated charge transport for non-radicals. In contrast, the PSD of Mono-PFPR, Di-PFPR, Di-PFNR and Di-NFNR scales as *G*^1.4^, *G*^1.6^, *G*^1.2^ and *G*^1.9^ (the *n* of *G*^*n*^ is the scaling exponent), respectively, suggesting that both through-bond and through-space coupling participate in the charge transport but to different degrees. The scaling exponent of 1.9 for Di-NFNR implies that the through-space coupling dominates the charge transport, in agreement with the highest 3,3′/12,12′interference which limits the through-bond transport facilitating the through-space channel. However, Di-PFNR has a lower scaling exponent of 1.2, in agreement with its highest molecular conductance, as the diminished inter-quinoidal interference favours maximal through-bond charge transport.

**Fig. 3:**
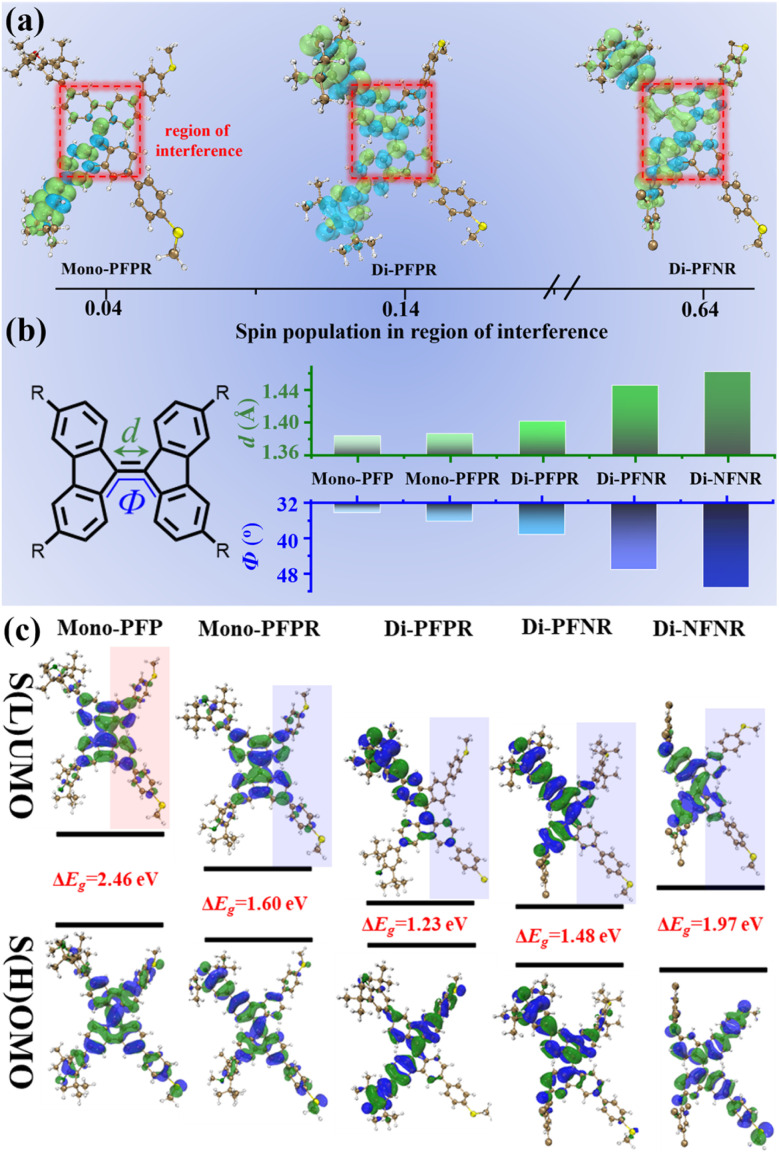
(a) The calculated spin density distribution of the ground state (S_0_) of Mono-PFPR, Di-PFPR and Di-PFNR (with iso 0.001), highlighted in red are the spin population in the region of interference, shown as insets. (b) The theoretical calculation of the torsion angle *Φ* (green) and C=C double bond length (*d*) (blue) for Mono-PFP, Mono-PFPR, Di-PFPR, Di-PFNR and Di-NFNR. (c) The calculated spatial distribution of orbital levels of Mono-PFP, Mono-PFPR, Di-PFPR, Di-PFNR and Di-NFNR (shaded part: conducting path).

We have carried out quantum-chemical calculations^38^ by utilizing the Gaussian16 software to optimize molecular configurations and investigate the impact of radical injection on the molecular structure first, followed by the exploration of its influence on electrically conductive properties. First, the spin populations in the region of interference on the 9,9′-BF skeletons (highlighted in the red boxes) are calculated to be 4%, 14%, and 64% for Mono-PFPR, Di-PFPR and Di-PFNR, respectively (Fig. 3a). The gradual increase of the spin population in the order of Mono-PFPR, Di-PFPR and Di-PFNR suggests that the impact of radical electrons on the 9,9′-BF skeleton increases gradually in this way. For the non-radical compound, the 9,9′-BF core structure is unaffected by spin (0% spin population).

As shown in Fig. 3b and S18,† a progression from a non-radical (Mono-PFP) to a mono-radical (Mono-PFPR) to diradicals (Di-PFPR and Di-PFNR), and finally to a quinone-like structure (Di-NFNR) can be monitored by the evolution of the C=C double bond lengths of the radicals which respectively changed from 1.383 to 1.386, 1.401, 1.445, and eventually to 1.462 Å. Furthermore, for the same compounds, the twist angle *Φ* of the adjacent π planes varied from 34° to 36°, 39°, 47°, and 51°. The reduction in double-bond character implies a decrease in electron cloud overlap, which may reduce the electron transport efficiency. Meanwhile, the increased torsion angle could weaken the electronic coupling between the π-electron systems, further lowering the electron transport efficiency and opening the through-space channel which is observed in some cases. These structural changes contribute to the decrease in single-molecule conductance with the introduction of radicals. Simultaneously, the introduction of radicals into Mono-PFPR, Di-PFPR, Di-PFNR and Di-NFNR brings about a net localization of electron distribution in the conducting path compared to the precursor (Fig. 3c), with both features resulting from the cross-conjugation effect and imparting the experimentally observed decrease in the electrical transport capacity.

**Fig. 4:**
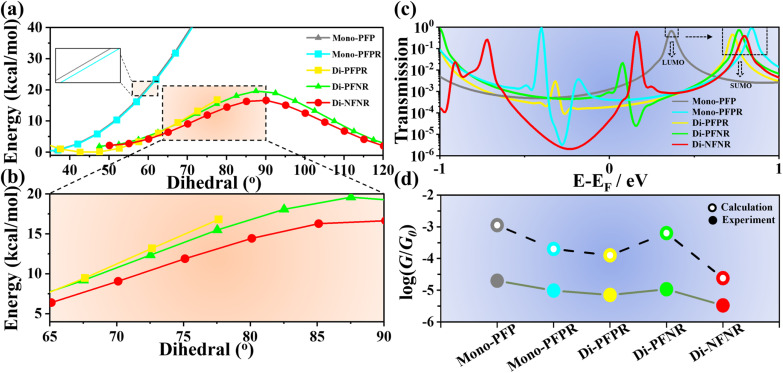
(a) Theoretical calculation of the rotational barriers for Mono-PFP, Mono-PFPR, Di-PFPR and Di-PFNR. (b) Local magnification of the selected region in (a). (c) Transmission spectra, plotted semi-logarithmically *vs.* energy relative to the Fermi energy, for Mono-PFP, Mono-PFPR, Di-PFPR, Di-PFNR and Di-NFNR. (d) Comparisons of calculated and measured conductance of the selected compounds.

In addition, using the room temperature barrier of 20 kcal mol^−1^ as a reference, the rotational barriers of Mono-PFPR, Di-PFPR, Di-PFNR, and Di-NFNR are lower than those of the non-radicals Mono-PFP, Di-PFP, Di-PFN, and Di-NFN (Fig. 4a) indicating that radical injection and delocalization reduce the central double bond character and consequently reduce the torsional potential energy, making the bending/torsion process easier to occur. Larger twisting impedes the formation of the quinoidal path in the conducting state thus revealing an inverse relationship between the molecular torsion angle and the STM-BJ experimental single-molecule conductance.

We utilized the Quantum ATK software (version Q2019.12-SP1) to simulate the charge transport properties of the studied molecules (Fig. 4c and S19†).^39^ First, we optimized the molecular geometries of the radicals and closed-shell compounds using Gaussian16 software. The calculated transmission spectra in Fig. S19† show that there is very little difference in the transmission near the Fermi level for the non-radical molecules (Mono-PFP, Di-PFP, Di-PFN, and Di-NFN) owing to the negligible difference imparted by the substituent groups on their closed-shell π-electronic configurations. For radicals, a small resonance + antiresonance structure can be seen in the radicals, produced by bound states (*i.e.*, of the quasi-free unpaired electron) in the continuum (*i.e.*, by the inter-SMe conductance channel). Furthermore, for mono- and diradicals, the singly unoccupied molecular orbital (SUMO) contributes significantly to the transmission, which is energetically far away from the *E*_F_ than the lowest unoccupied molecular orbital (LUMO) of the non-radical Mono-PFP. This results in lower conductance values for the radical compounds compared to their non-radical counterparts. In Di-NFNR, we observed a transmission dip (characteristic of destructive interference) at an energy of −0.22 eV [relative to the density functional theory (DFT-predicted Fermi energy)] in the transmission spectrum (Fig. 4c), which is in agreement with the largest interference for the conducting paths, therefore yielding the lowest molecular conductance.

In summary, we have demonstrated that the introduction of radicals into 9,9′-BF derivatives significantly influences their electrical transport properties at the single-molecule level. Our findings reveal that intramolecular spin delocalization, facilitated by radical and diradical species, can modulate the conducting pathways from linear conjugation to cross-conjugation. This modulation results in a substantial reduction in single-molecule conductance. Specifically, the DFT results reveal that the C=C double bond length monotonically increases from 1.383 Å to 1.462 Å, and the twist angle of the adjacent π planes gradually increases from 34° to 51° with the introduction of the monoradical (Mono-PFPR), diradical (Di-PFPR) and finally the formation of a quinone-like structure (Di-NFNR), leading to the experimentally observed decrease in the electrical transport capacity. Through systematic design and synthesis of 9,9′-BF derivatives with various diradical characters, we have elucidated the relationship between spin delocalization and molecular conductance. The *y*_0_ parameter was found to be a crucial parameter in quantifying the extent of spin–spin coupling and its impact on the molecular skeleton. Our quantum transport simulations and experimental measurements using STM-BJ techniques further support these conclusions, showcasing the unique spin-related electronic properties of these radical-based systems. The integration of single-molecule techniques with radical chemistry offers new insights into the structure–function relationships of organic radicals.

## Data availability

The data supporting this article have been included as part of the ESI.†

## Author contributions

Y. Z., D. W. and X. L. supervised the project. H. Z. carried out the synthetic work. Y. H. carried out the ESR studies. H. Z. and X. L. prepared the manuscript. S. M. Q. and J. C. contributed to the electronic structure analysis. H. Z. and W. H. carried out the computational studies. All authors discussed the results and commented on the manuscript.

## Conflicts of interest

The authors declare no competing financial interest.

## Supplementary Material

SC-016-D4SC07256A-s001
