# Supplementary material for: Radical-induced single-molecule conductance tuning in 9,9′-bifluorenylidene derivatives
Source: Chem Sci. 2025 Feb 10;16(12):5099–108. doi: 10.1039/d4sc07256a (PMC11833457; doi:10.1039/d4sc07256a)

Supplementary Information (SI) for Chemical Science.
This journal is © The Royal Society of Chemistry 2025

Supporting Information for

Radical-Induced Single-Molecule Conductance Tuning in 9,9'-Bifluorenylidene Derivative

Hanjun Zhang,[a] Lichuan Chen,[b] Yunzhu Huang,[a] Xiaodong Liu,*[a] Sergio Moles Quintero,[c] Wenjing Hong,[b] Dongsheng Wang,*[a] Juan Casado [c] and Yonghao Zheng *[a, d]

[a].School of Optoelectronic Science and Engineering, University of Electronic Science and Technology of China (UESTC), Chengdu 611731, People's Republic of China..
[b].State Key Laboratory of Physical Chemistry of Solid Surfaces College of Chemistry and Chemical Engineering Xiamen University Xiamen, Xiamen 361005, People's Republic of China.
[c].Department of Physical Chemistry, University of Málaga, Campus de Teatinos s/n, Málaga 29071, Spain.
[d].State Key Laboratory of Organic Electronics and Information Displays & Institute of Advanced Materials (IAM), Nanjing University of Posts & Telecommunications, 9 Wenyuan Road, Nanjing 210023, People's Republic of China.
Yonghao Zheng, Xiaodong Liu and Dongsheng Wang
Email: zhengyonghao@uestc.edu.cn (Y.Z.); xdliu@uestc.edu.cn (X.L.); wangds@uestc.edu.cn (D.W.)

1. General information.

All reagents were purchased from Bide Pharmatech Ltd. and Adamas and used as received. Flash column chromatography was performed with Haiyang silica gel (200-300 mesh). All reaction mixtures and column eluents were monitored by thin layer chromatography (TLC) using commercial Huanghai glass plates (HSGF 254, 2.5 x 8 cm). The plates were visualized under UV radiation at 254 and 365 nm. UV-*Vis*-NIR absorption spectra were recorded with a UV-Vis spectrophotometer (Shimadzu, UV-2600). The solutions with a concentration of 0.1 mmol/L in dichloromethane (DCM) were taken for absorption measurement. MALDI-TOF mass spectrums were recorded with a Matrix-assisted laser desorption/ionization reflection time-of-flight mass spectrometer (Shimadzu, AXIMA Confidence, Matrix: 7,7,8,8-tetracyanoquinodimethane (TCNQ)). High resolution mass spectra (HRMS) were recorded on a Waters-Q-TOF-Premier (ESI). Nuclear magnetic resonance (NMR) spectra were obtained on a Bruker AV II-400 MHz spectrometer. Due to the limitations of the 400 MHz NMR instrument we used, some peaks may overlap with each other or merge together, making the proton assignments for these peaks difficult. Therefore, we assigned these overlapping or merging peaks to multiple proton signals. ESR spectroscopy measurement was conducted by a Bruker EMX plus X-band spectrometer with 9.8 GHz microwave frequency. The sample for ESR measurement with a concentration of 0.1 mmol/L in anhydrous toluene or solid solution. Cyclic voltammograms were measured on a Shanghai Chenhua CHI 660E electrochemical workstation. In this work, we used a home-built Scanning Tunneling Microscope Break Junction (STM-BJ) set-up to perform the single-molecule conductance measurement. The naming convention for the compounds in this paper is as follows: Mono-PFPR stands for "Mono-radical Phenoxy Fluorenylidene Radical"; Di-PFPR stands for "Di-radical Phenoxy Fluorenylidene Radical". "Mono" indicates the presence of one radical, while "Di" indicates the presence of two radicals. "P" stands for the Phenoxy group in the molecule. "F" denotes Fluorenylidene (a fluorene-based structure with a double bond); "R" signifies the radical component of the molecule.

2. Synthesis and Characterization.

Synthesis of T1

4,4,5,5-tetramethyl-2-(4-(methylthio)phenyl)-1,3,2-dioxaborolane (5.0 g, 20 mmol), 3,6-dibromo-9H-fluoren-9-one (13.4 g, 40 mmol), $Pd(PPh_3)_4$ (2.2 g, 2 mmol) and Na_2CO_3 (8.4 g, 80 mmol) was added into a 1000 mL round-bottom flask with 500 mL of a 1:5 (v/v) THF/H_2O solvent mixture under N_2 atmosphere. The mixture was heated at 90 °C and stirred overnight. The mixture was cooled to room temperature, followed by the addition of 100 mL of water. The mixture was then extracted with dichloromethane (DCM) (3 × 100 mL). The collected organic layers were washed with water, dried over anhydrous Mg_2SO_4 and the solvent was removed under reduced pressure, and purified by column chromatography on silica gel (eluent: 3:1 (v/v) hexane/DCM). Recrystallized from DCM/ hexane to obtain yellow solid **T1**: 4.5 g, 60 % yield. ^{1}H NMR (400 MHz, Chloroform-*d*) 7.76 - 7.65 (m, 3H), 7.66 (d, J = 1.5 Hz, 1H), 7.60 - 7.49 (m, 4H), 7.46 (d, J = 1.3 Hz, 1H), 7.36 (d, J = 2.0 Hz, 1H), 2.55 (s, 3H). ^{13}C NMR (101 MHz, Chloroform-*d*) 192.1, 147.3, 145.8, 144.0, 139.8, 136.4, 133.4, 132.9, 132.2, 129.6, 128.1, 127.5, 125.1, 123.9, 119.0, 15.6. MALDI-TOF m/z: $[M]^+$ calcd. for $C_{20}H_{13}BrOS$: 379.9870, found 379.0769.

Synthesis of T2

T1 (1.9 g, 5 mmol), 2,6-di-tert-butyl-4-(4,4,5,5-tetramethyl-1,3,2-dioxaborolan-2-yl)phenol (1.7 g, 5 mmol), $Pd(PPh_3)_4$ (0.6 g, 0.5 mmol) and Na_2CO_3 (2.1 g, 20 mmol) was added into a 500 mL round-bottom flask with 250 mL of a 1:5 (v/v) THF / H_2O solvent mixture under N_2 atmosphere. The mixture was heated at 90 °C and stirred overnight. The mixture was cooled to room temperature, followed by the addition of 100 mL of water. The mixture was then extracted with DCM (3 × 50 mL). The collected organic layers were washed with water, dried over anhydrous Mg_2SO_4 and the solvent was removed under reduced pressure, and purified by column chromatography on silica gel (eluent: 2:1 (v/v) hexane/DCM). Recrystallized from DCM/ hexane to obtain yellow solid **T2**: 1.0 g, 40 % yield. ^{1}H NMR (400 MHz, Chloroform-*d*) 7.70 - 7.61 (m, 4H), 7.54 (d, J = 1.8 Hz, 2H), 7.42 (d, J = 2.1 Hz, 2H), 7.33 - 7.16 (m, 4H), 5.33 (s, 1H), 2.47 (s, 3H), 1.24 - 1.14 (s, 18H). ^{13}C NMR (101 MHz, Chloroform-*d*) 192.1, 153.5, 148.0, 146.0, 138.36, 135.9, 135.5, 132.6, 126.8, 126.6, 125.7, 125.6, 123.7, 117.8, 117.7, 30.9, 29.3, 14.6. MALDI-TOF m/z: $[M]^+$ calcd. for $C_{34}H_{34}O_2S$: 506.2280, found 506.1136.

Synthesis of T3

T1 (1.9 g, 5 mmol), 2,4,6-trichloroaniline (2.9 g, 15 mmol), Tris (dibenzylideneacetone) dipalladium (0.69 g, 0.75 mmol), 2,2'-Bis (diphenylphosphino) -1,1'- binaphthalene (1.4 g, 2.25 mmol) and sodium tert-butoxide (1.4 g, 15 mml) was added into a 500 mL round-bottom flask with 250 mL anhydrous toluene under N_2 atmosphere. The mixture was heated at 120 ^{o}C and stirred for six hours. The mixture was cooled to room temperature, and the solvent was removed under reduced pressure, and purified by column chromatography on silica gel (eluent: 1:1 (v/v) hexane/DCM). Recrystallized from DCM/hexane to obtain yellow solid **T3**: 1.5 g, 60 % yield. ^{1}H NMR (400 MHz, Chloroform-*d*) 7.58 (d, *J* = 2.7 Hz, 2H), 7.53 - 7.44 (m, 5H), 7.39 (m, 3H), 7.28 - 7.24 (m, 2H), 5.96 (s, 1H), 2.45 (s, 3H). ^{13}C NMR (101 MHz, Chloroform-*d*) 190.9, 148.4, 145.5, 145.3, 143.0, 138.2, 135.9, 133.3, 132.6, 132.5, 131.0, 128.0, 126.5, 126.2, 125.7, 125.1, 123.2, 117.5, 113.4, 105.7, 28,7. MALDI-TOF m/z: $[M]^+$ calcd. for $C_{26}H_{16}Cl_3NOS$: 495.0018, found 495.9092.

Synthesis of Di-PFP, **Di-PFN** and **Di-NFN**

A mixture of **T2** (1.0 g, 2 mmol), **T3** (1.0 g, 2 mmol), and Lawesson's reagent (0.81 g, 2 mmol) in toluene anhydrous (50 mL) was refluxed under N_2 atmosphere for 12 h, upon cooling to room temperature, the reaction mixture was extracted from DCM, and the solvent was removed under reduced pressure, and purified by column chromatography on silica gel (eluent: 1:1 (v/v) hexane/DCM). Recrystallized from DCM/hexane to obtain red solid **Di-PFP**: 0.39 g, 20 % yield, **Di-PFN**: 0.19 g, 10 % yield and **Di-NFN**: 0.38 g, 20 % yield.

Di-PFP: ^{1}H NMR (400 MHz, Chloroform-*d*) 7.92 - 7.83 (m, 4H), 7.61 (d, *J* = 6.2 Hz, 4H), 7.59 - 7.55 (m, 4H), 7.46 (s, 4H), 7.30 (d, *J* = 6.1 Hz, 4H), 7.17 (s, 2H), 5.27 (s, 2H), 2.47 (s, 6H), 1.47 (s, 36H). ^{13}C NMR (101 MHz, Chloroform-*d*) 154.0, 143.2, 141.5, 138.2, 136.4, 132.4, 127.5, 127.1, 127.0, 126.0, 125.5, 124.0, 118.2, 34.6, 30.4, 15.9. MALDI-TOF m/z: $[M]^+$ calcd. for $C_{68}H_{68}O_2S_2$: 980.4661, found 980.2998.

Di-PFN: ^{1}H NMR (400 MHz, Chloroform-*d*) δ 8.40 (d, *J* = 4.5 Hz, 3H), 8.32 -8.22 (m, 1H), 8.07 - 7.87 (m, 2H), 7.76 (s, 1H), 7.73 - 7.56 (m, 5H), 7.58 - 7.26 (m, 12H), 6.59 (d, *J* = 4.4 Hz, 1H), 5.31 (d, *J* = 3.9 Hz, 1H), 2.54 (d, *J* = 2.8 Hz, 6H), 1.54 (s, 18H). ^{13}C NMR (101 MHz, Chloroform-*d*) δ 154.7, 142.7, 141.3, 141.0, 140.0, 138.6, 137.7, 136.4, 134.4, 130.6, 129.0, 127.4, 127.3, 127.0, 126.9, 124.0, 118.1, 113.5, 34.6, 29.7, 15.9. MALDI-TOF m/z: $[M]^+$ calcd. for $C_{60}H_{50}Cl_3NOS_2$: 971.5360, found 971.1285.

Di-NFN: ^{1}H NMR (400 MHz, Chloroform-*d*) 8.28 (d, *J* = 4.3 Hz, 2H), 8.13 (d, *J* = 3.5 Hz, 2H), 7.69 (d, *J* = 1.7 Hz, 2H), 7.54 (d, *J* = 3.6 Hz, 4H), 7.38 - 7.25 (m, 10H), 6.92 (d, *J* = 2.6 Hz, 2H), 6.51 (d, *J* = 3.4 Hz, 2H), 5.87 (s, 2H), 2.46 (s, 6H). ^{13}C NMR (101 MHz, Chloroform-*d*) 145.6, 142.6, 141.2, 140.5,137.3, 137.2, 133.9, 132.7, 131.1, 128.0, 126.4, 125.9, 123.8, 117.0, 113.9, 14.9. MALDI-TOF m/z: $[M]^+$ calcd. for $C_{52}H_{32}Cl_6N_2S_2$: 958.0138, found 958.0311.

Synthesis of Mono-PFP

Under an atmosphere of N_2, **Di-PFP** (0.2 g, 0.2mmol) in degassed DMF (50 mL) was successively added barite (0.082 g, 0.26 mmol) and CH_3I (1 mL, 0.65 mmol). The reaction mixture was stirred overnight. Et_2O (100 mL) was then added. The organic layer was extracted with H_2O (100 mL), aqueous NaOH (1 M, 150 mL) and H_2O (2×250 mL). The combined extracts were dried with $MgSO_4$ and concentrated in vacuo and purified by column chromatography on silica gel (eluent: 1:1 (v/v) hexane/DCM). Recrystallized from DCM/hexane to obtain red solid **Mono-PFP**: 0.21 g, 90 % yield. ^{1}H NMR (400 MHz, Chloroform-*d*) 8.42 - 8.34 (m, 4H), 7.93 - 7.82 (m, 4H), 7.65 - 7.56 (m, 4H), 7.49 (d, *J* = 5.4 Hz, 4H), 7.38 (d, *J* = 4.4 Hz, 4H), 7.34 - 7.27 (m, 4H), 5.27 (s, 1H), 3.69 (s, 3H), 2.47 (s, 6H), 1.46 (d, *J* = 3.0 Hz, 36H). ^{13}C NMR (101 MHz, Chloroform-*d*) δ 158.6, 143.1, 141.8, 138.9, 137.2, 136.9, 136.8, 135.3, 131.3, 126.4, 125.9, 125.1, 125.0, 124.5, 123.5, 123.0, 117.3, 117.1, 63.4, 35.0, 33.5, 31.2, 14.8. MALDI-TOF m/z: $[M]^+$ calcd. for $C_{69}H_{70}O_2S_2$: 994.4817, found 994.3685.

Synthesis of Mono-PFPR

PbO_2 (4 mmol) and **Mono-PFP** (0.1 mmol) were stirred in DCM (10 mL) for 1 minute, the excess PbO_2 was filtered off and the solvent was removed under reduced pressure to yield **Mono-PFPR** (85%) as a black solid. **Mono-PFPR**: MALDI-TOF m/z: $[M]^+$ calcd. for $C_{69}H_{69}O_2S_2$: 993.4739, found 993.4309; HRMS (ESI^+): calcd. For $C_{69}H_{69}O_2S_2$: 993.4739, found $[M]^+$ 993.4817.

Synthesis of Di-PFPR, Di-PFNR and **Di-NFNR**
The synthesis process **Di-PFPR, Di-PFNR** and **Di-NFNR** are like **Mono-PFPR**, with yields of 85%, 85% and 90%, respectively.

Di-PFPR: MALDI-TOF m/z: $[M]^+$ calcd. for $C_{68}H_{66}O_2S_2$: 978.4504, found 978.3843; HRMS (ESI^+): calcd. For $C_{68}H_{66}O_2S_2$: 978.4504, found $[M]^+$ 978.4663.

Di-PFNR: MALDI-TOF m/z: $[M]^+$ calcd. for $C_{60}H_{48}Cl_3NOS_2$: 969.5200, found 969.1370; HRMS (ESI^+): calcd. For $C_{60}H_{48}Cl_3NOS_2$: 969.5200, found $[M]^+$ 969.5365.

Di-NFNR: ^1H NMR (400 MHz, DMSO-*d6*) 8.45 (s, 2H), 8.34 - 8.30 (d, *J* = 2.6 Hz, 4H), 7.85 - 7.75 (m, 10H), 7.60 (s, 2H), 6.61 (s, 2H), 6.45 (d, *J* = 2.9 Hz, 2H), 6.01(d, *J* = 3.8 Hz 2H). (Due to its poor solubility, only its ^1H NMR spectrum was obtained) MALDI-TOF m/z: $[M]^+$ calcd. for $C_{52}H_{30}Cl_6N_2S_2$: 955.9982, found 955.8721; HRMS (ESI^+): calcd. For $C_{52}H_{30}Cl_6N_2S_2$: 955.9982, found $[M]^+$ 956.0142.

Synthesis of 4FR and **4NR**
Synthetic steps like **Di-PFPR**.

4FR: MALDI-TOF m/z: $[M]^+$ calcd. for $C_{82}H_{92}O_4$: 1140.6996, found 1140.5245.

4NR: MALDI-TOF m/z: $[M]^+$ calcd. for $C_{50}H_{22}Cl_{12}N_4$: 1104.1540, found 1104.2127.

***3. cis–trans* isomerization.**
The reaction of **T2** and **T3** with Lawesson's reagent gave the possiable mixture of *cis* and *trans* isomers. These isomers were not isolable. Therefore, we took **Mono-PFP** moleucle as an example to study in Nuclear Magnetic Resonance (NMR) spectroscopy, The presence of stereoisomers (*cis* and *trans*) is typically confirmed by observing the splitting patterns of peaks in the range of 7-9 ppm. In the absence of structural isomers, a single peak should be observed at position "a_n" (n = 1, 2, 3 or 4), as shown in **Fig. S5**. However, we observed two doublets with chemical shifts at 7.78 and 7.86 ppm, which can be attributed to the phenyl ring protons at the "a_1/a_3" and "a_2/a_4" positions, respectively. This phenomenon suggests the presence of structural isomers. Moreover, we note variations in the nuclear magnetic resonance signals as the temperature increases, which also indicates the presence of structural isomers. Utilizing 2D-NOESY spectroscopy, the peak at "b/d" positions shows coupling with the peaks at "h" and "i" positions, confirming the presence of the trans configuration. On the other hand, we only observed the conductance for *cis* isomer in the STM-BJ. The *trans* isomer is too long and with very low conductance for the Scanning Tunneling Microscope Break Junction (STM-BJ) measurement.

4. Additional Conductance Data.
Scanning Tunneling Microscope Break Junction. In this work, we used a home-built STM-BJ set-up to perform the single-molecule conductance measurement. A gold tip is used as a tip, which is fixed onto a piezo, while the piezo is adhered to the bottom of a stepping motor. The gold-plated silicon wafers that were used as the substrates were pretreated by piranha lotion (98% H_2SO_4: 30% H_2O_2 = 3:1, v/v) followed by washing with ultra-pure water (18.25 MΩ·cm, Qianhe QH-DI-15) before each experiment, and then dried in a vacuum oven. After dropping 5 μL target solution (All sample concentrations were 0.1 mmol/L in anhydrous TCB) onto the substrate, a single-molecule was trapped at the periodic break junction to form single-molecule junction. Our group's STM-BJ used for testing has a detection limit at $G > 10^{-6}\ G_0$.

Statistical Analysis
The molecular conductance data, including typical individual conductance traces, 1D and 2D conductance-displacement histograms, are analyzed with the homemade single-molecule break junctions experimental data processing software built with Python and PyQt5, XMe DataAnalysis, which is available for research users at https://github.com/Pilab-XMU/XMe_DataAnalysis.[1]

Conductance measurements individual conductance traces were obtained by cutting the continuous conductance–time curve at high current limit of 50 μA and then collecting around 6000

data points with a sampling rate of 20 kHz as effective data in a single trace. The time axis is converted into Δ*z* axis by multiplying the sampling frequency and the moving rate of the tip.[2]

In 1D conductance histograms, the conductance axis was divided into 1100 bins with log (G/G_0) from -10 to 1, and when the discrete conductance points fell within one bin, the count of the bin added 1. The histograms were constructed by accumulating all the data points with conductance as x axis and counts as y axis. The peak shift in a conductance histogram was obtained by Gaussian fitting, which represents the most probable molecular conductance. 2D conductance-displacement histograms were plotted by overlapping the conductance traces with a bin size of 1100 for log (G/G_0) from -10 to 1, and 1000 for Δ*z* from -0.5 to 1.5 nm. All the traces are aligned with a relative zero point (Δz = 0) at log (G/G_0) = -0.3. Afterward, the 2D conductance-displacement histograms were obtained by drawing the data counts in each bin. The sample size gathered for the construction of each histogram is given in corresponding figure captions. We conducted three rounds of measurements on different batches of samples.

Flicker noise measurements.

To understand the electron tunneling mechanism in molecular junction, the noise power spectral density (PSD) analysis was employed to get insights into the transport mechanism in molecular junctions.[3] For the conductance noise measurement, the tip was controlled to stabilize for 150 ms after being removed from the substrate surface and fishing a molecule to form the molecular junction. To get the PSD, we cut out the conductance curve to perform discrete Fourier transformation, and the conductance data were squared. The 2D histogram of normalized flicker noise power versus average conductance was constructed from thousands of conductance traces. The 2D Gaussian distribution fitting was applied to determine the scaling power.[4] The conductance power was normalized from $G^{1.0}$ to $G^{2.0}$, and the correlation power was determined by the correlation coefficient, which showed the smallest absolute value in the fitted Gaussian distribution equations.

The value of PSD was determined by the correlation between the average conductance and noise power. Through-space and through-bond coupling are fundamental concepts to understand electronic coupling, which also determine the electronic properties of the single-molecule junction. In the former, the hybridization between molecular orbitals and electronic states of the metal electrode occurs through a chemical bond, while in the latter, orbitals responsible for charge transfer do not participate in specific bond formation. When the through-bond coupling dominates the charge transport through the single molecule junction, the value would be 1.0. This means when the noise power is normalized by $G^{1.0}$ (G is the average conductance), there is no correlation between the noise power and G, leading to an orthogonal noise distribution. In contrast, when the through-space coupling is dominating, the value would be 2.0. This means that even when the noise power is normalized by $G^{1.0}$, there is still a strong correlation between the noise power and the average conductance. Thus, the noise distribution is a slant ellipse, suggesting that a larger conductance would lead to larger noise power and vice versa. If the noise power is normalized between $G^{1.0}$ and $G^{2.0}$, the charge transport is dominated by both the through space coupling and the through-bond coupling.

DFT calculations

Theoretical calculations were performed on the all compounds with the Gaussian 16 program suite using the density functional theory (DFT) with the Becke's three-parameter hybrid exchange functional and the Lee-Yang-Parr correlation functional employing the 6-311G(d) basis set for all atoms. Full geometry optimizations were carried out at the UDFT/ M06-2X/6-311G(d) level, and the obtained stationary points were characterized by frequency calculations. The spin densities were illustrated using Multiwfn[5] and VMD[6].

The theoretical transport performances were investigated using the combined DFT and nonequilibrium Green's functions (NEGF) method in Quantum Atomistix Tool Kit (Quantum ATK, 2019.12 version) software package. The Perdew-Burke-Ernzerhof (PBE) formulation of the generalized gradient approximation (GGA) is adopted as the exchange and correlation functional. The double-zeta plus polarization (DZP) basis set is for all the atoms, except for Au atoms with a double-zeta (DZ) basis set, to save computational time. A mesh cutoff energy is set to be 200 Ry

and the optimization force tolerance of the molecular device is a 0.05 eV/Å. The transmission probability through the molecular junction can be obtained from the Landauer-like Equation.

Fig. S1. Synthetic routes for the target molecules. (i) $Pd(PPh_3)_4$, KOH, H_2O, toluene, 90 °C; (ii) $Pd(PPh_3)_4$, KOH, H_2O, Toluene, 90 °C; (iii) $Pd_2(dba)_3$, BINAP, Sodium *tert*-butoxide, toluene, 120 °C; (iv) Lawesson's reagent, toluene, 120 °C; (v) Copper powder; (vi) PbO_2, DCM, rt; (vii) CH_3I, DMF, rt.

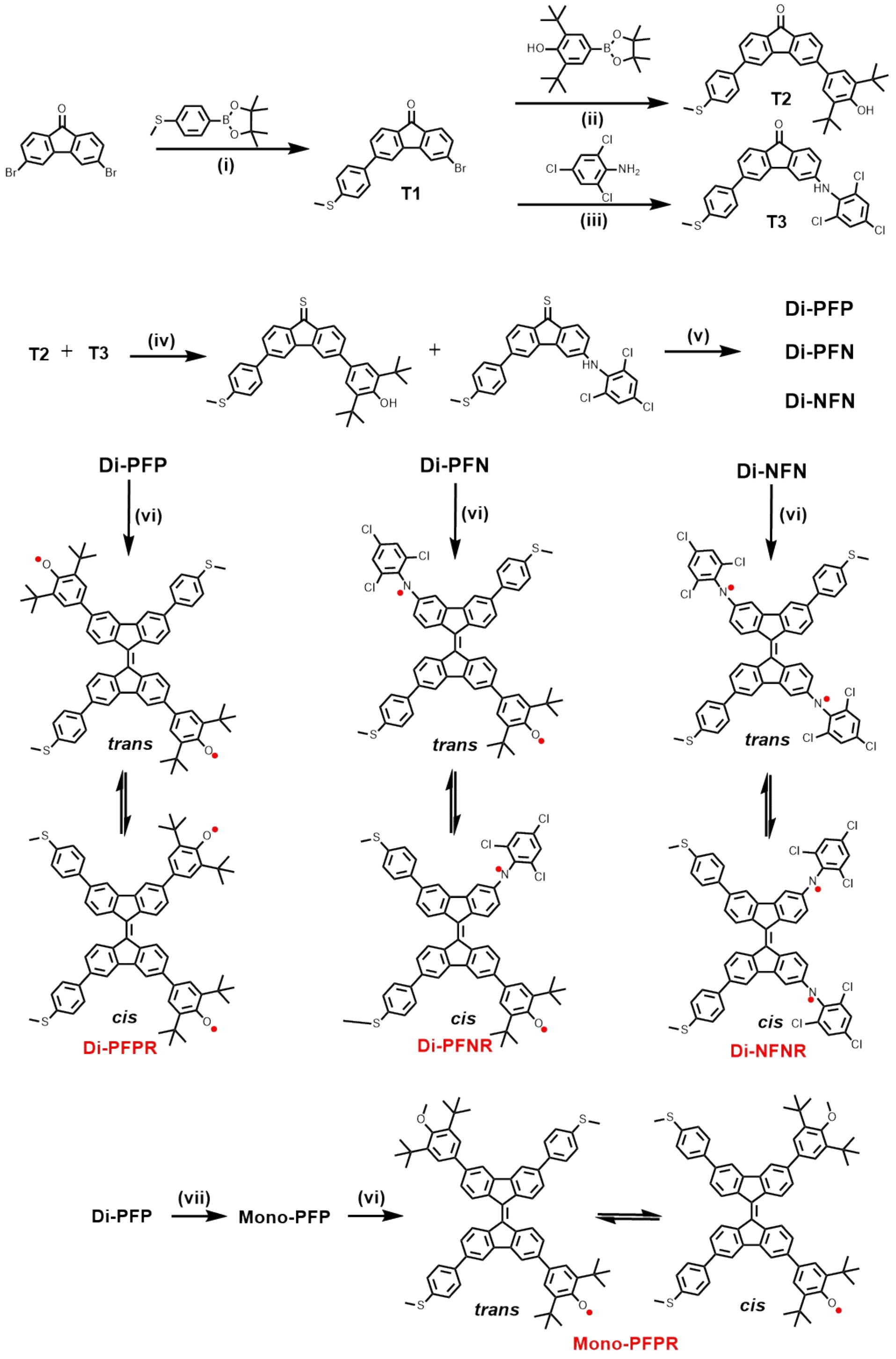

Fig. S2. The absorption spectra of **Mono-PFP** were compared with those of **Mono-PFP** irradiated with LED lights of (a) 365, (b) 470, and (c) 590 nm (20 mW/cm^2) in DCM at a concentration of 10^{-4} m in air for 120 min.

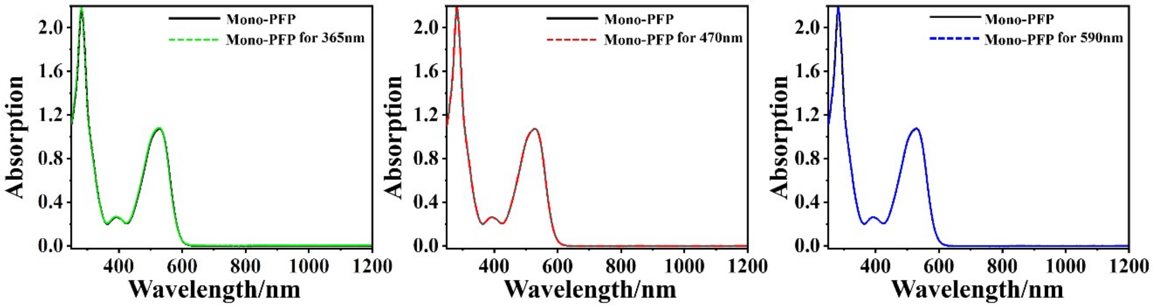

Fig. S3. The NMR spectra of **Mono-PFP** were compared with those of **Mono-PFP** irradiated with LED lights of (a) 365, (b) 470, and (c) 590 nm (20 mW/cm^2) in $CDCl_3$.

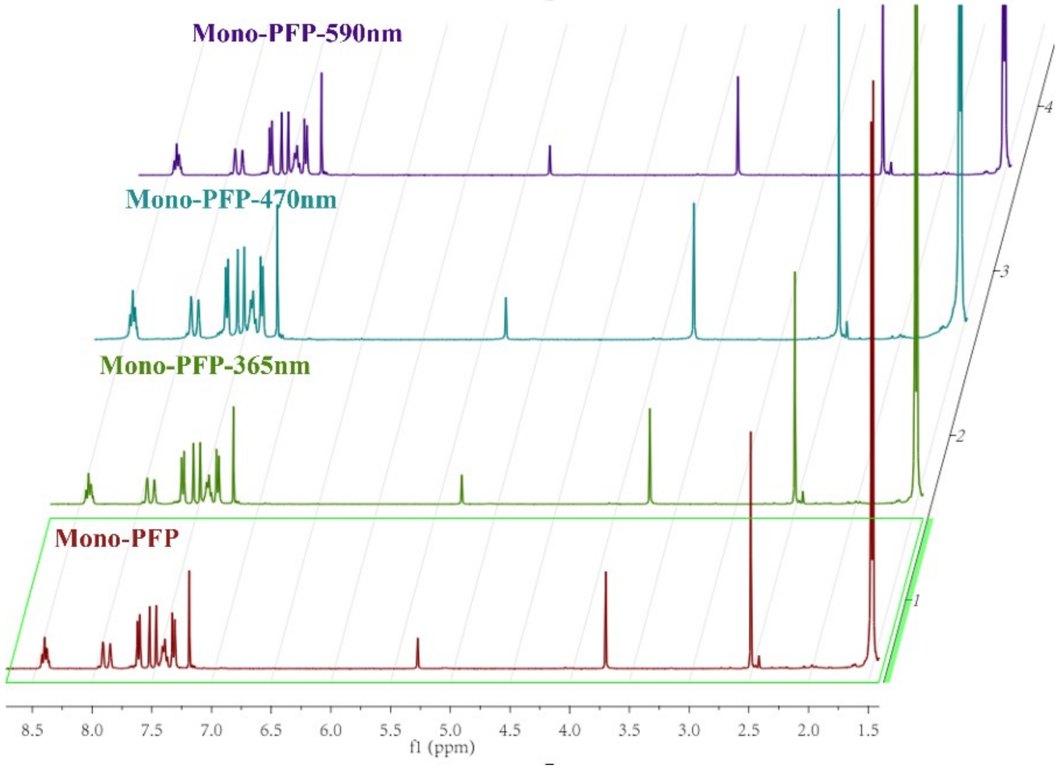

Fig. S4. Variable-temperature NMR spectra of **Mono-PFP** in toluene-d_8.

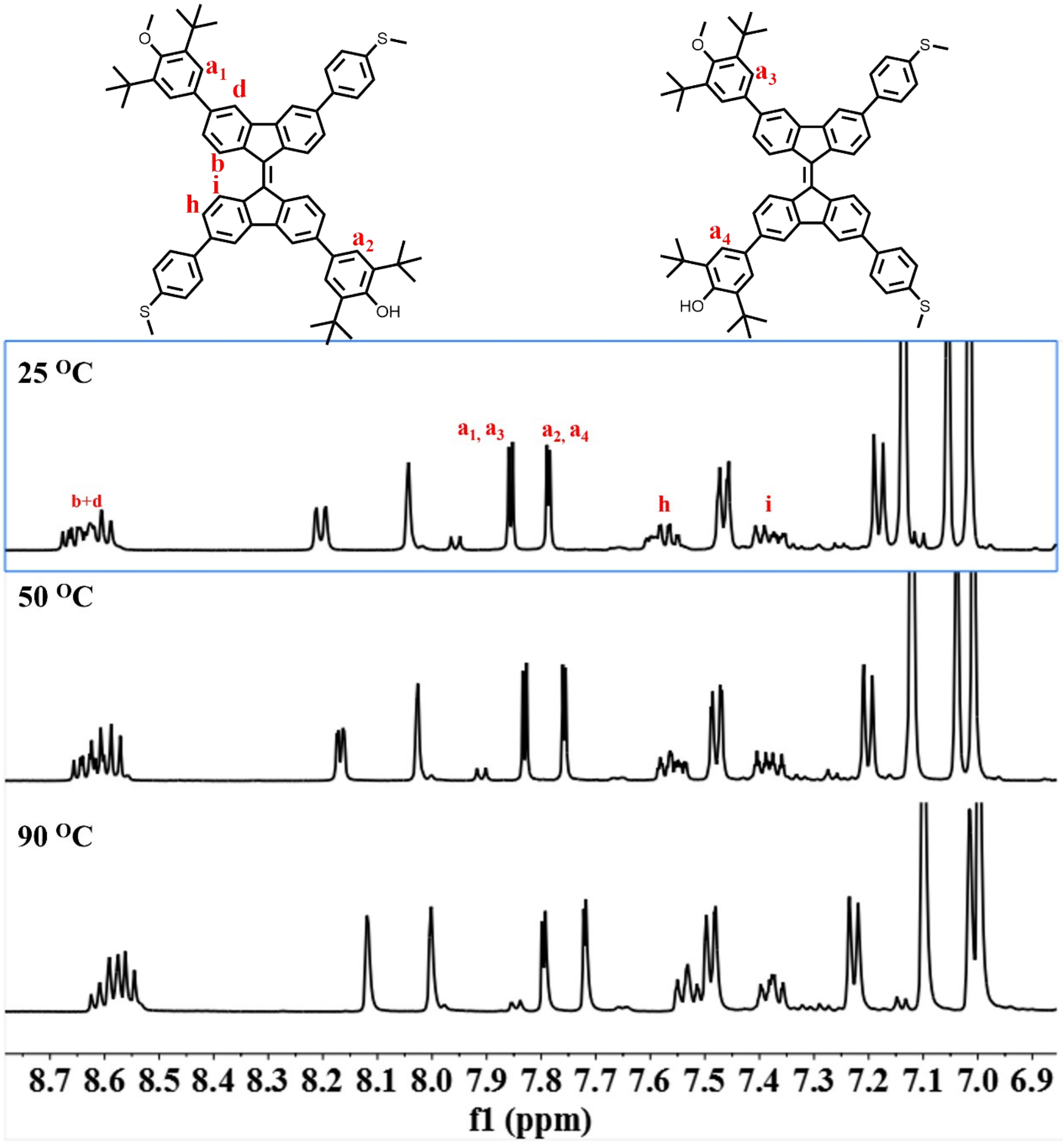

Fig. S5. 2D-NOESY of **Mono-PFP** in toluene-d_8. (a) Full spectrum and (b) locally spectrum.

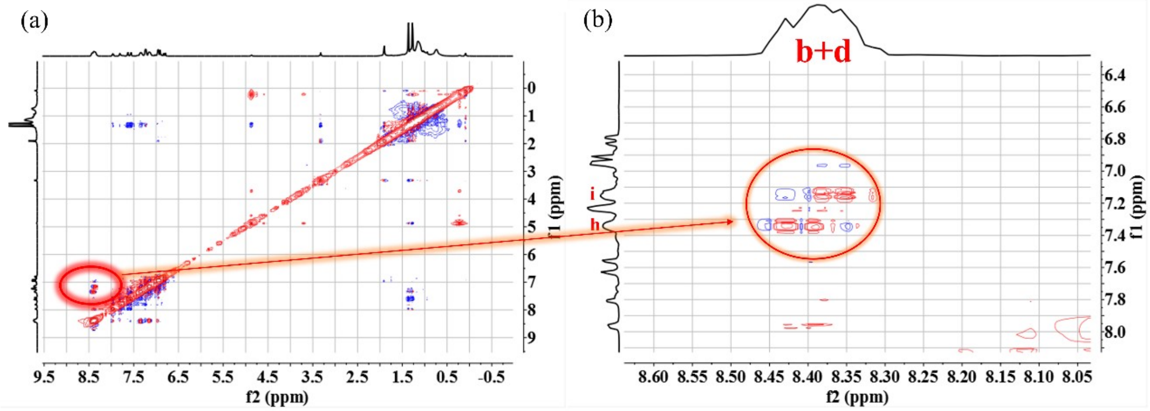

Fig. S6. ESR spectra of (a) **Mono-PFPR**, (b) **Di-PFPR** and (c) **Di-PFNR** and **Di-NFNR** in dry toluene.

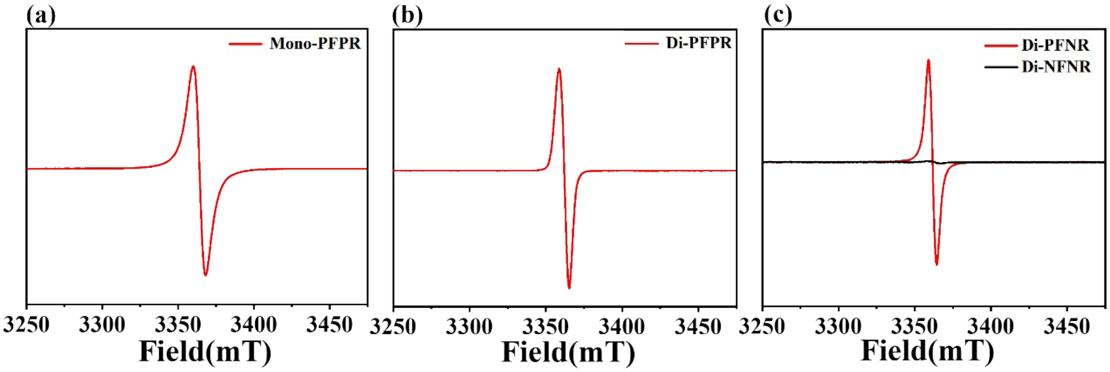

Fig. S7. *g*4 signal of ESR spectra. (a) **Mono-PFPR**, (b) **Di-PFPR** and (c) **Di-PFNR** at 140 K in dry toluene.

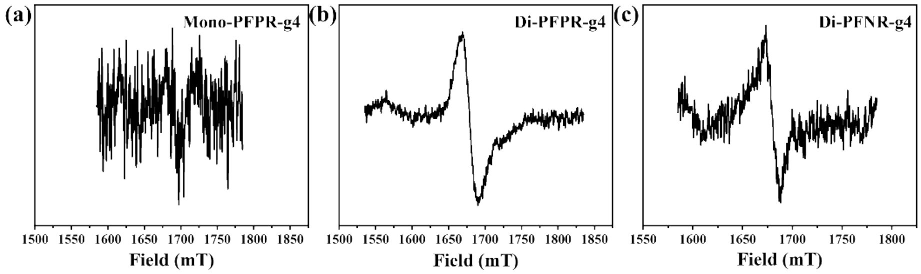

Fig. S8. The decay curves of UV-*vis*-NIR absorption spectra of **Mono-PFPR**, **Di-PFPR**, **Di-PFNR** and **Di-NFNR** in air, which can be fitted with the equation $I(t)=I_0(1/2)^{(t/\tau)}$ to determine the half-life (τ).

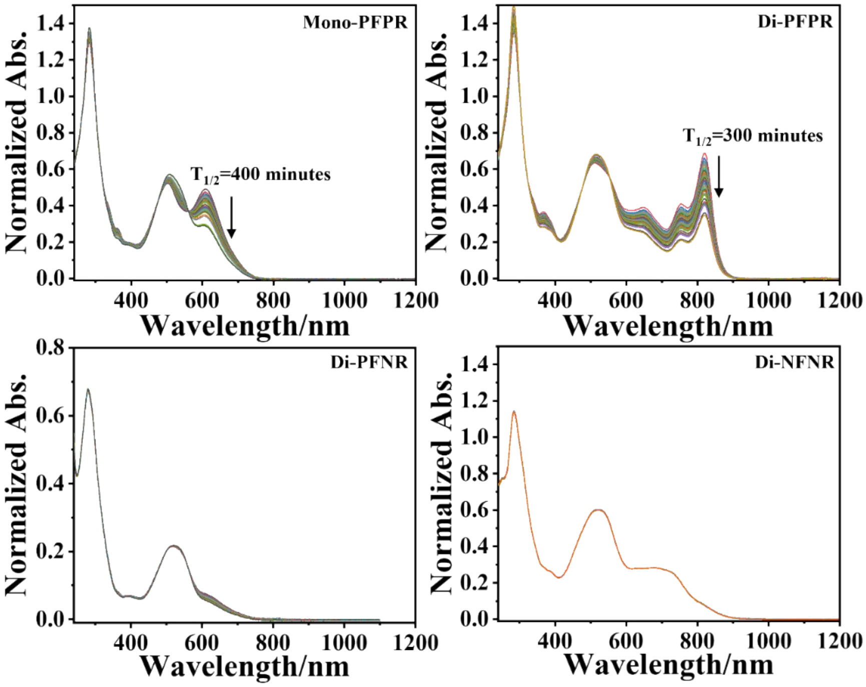

Fig. S9. 1D logarithmic conductance histograms of **Mono-PFP**, **Di-PFP**, **Di-PFN** and **Di-NFN**, pictured as insets. The insets show the relative-displacement distributions.

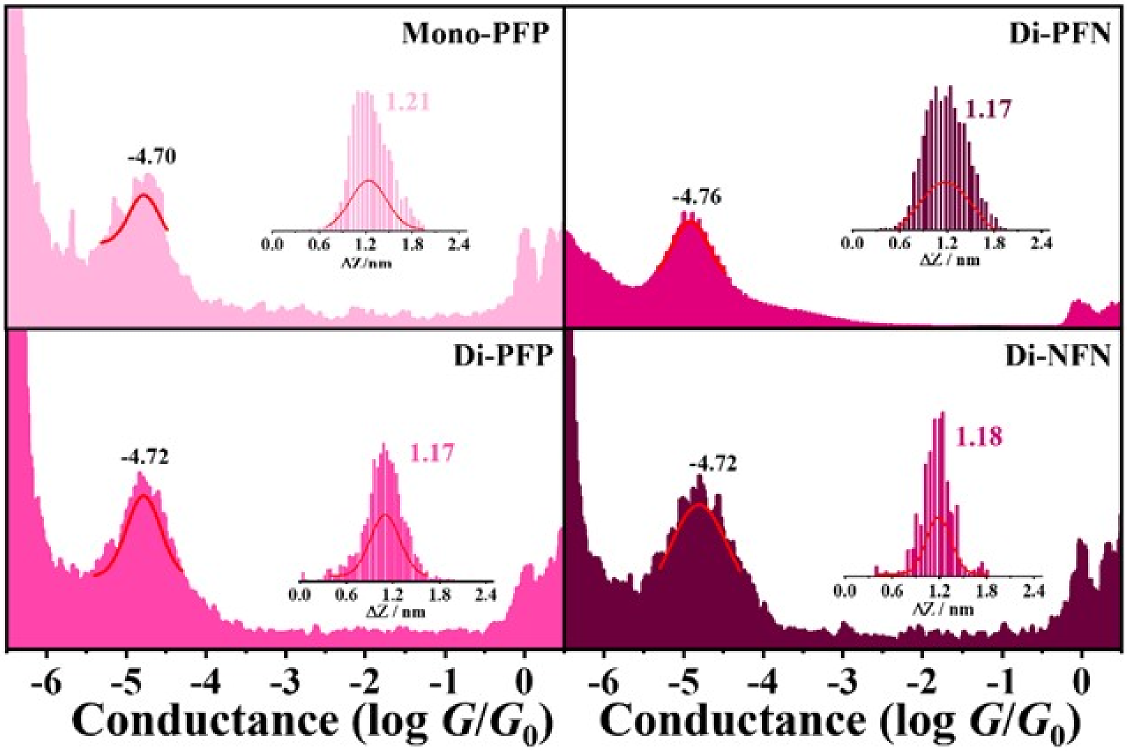

Fig. S10. The theoretically calculated molecular lengths of *cis* and *trans* isomer of 9,9'-BF derivatives.

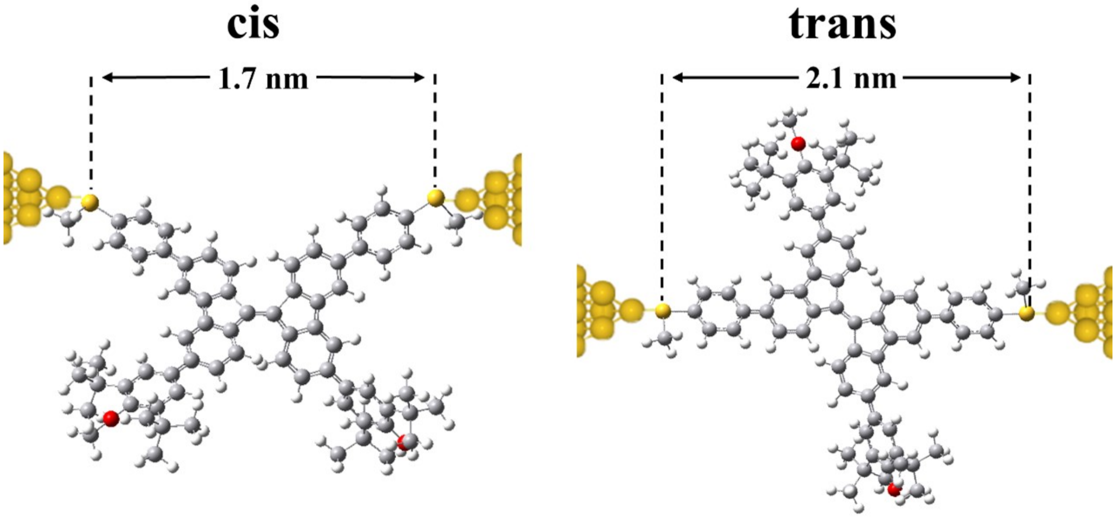

Fig. S11. Transmission spectra of plotted semi-logarithmically vs. energy relative to the Fermi energy for *cis* and trans isomer of **Mono-PFP**.

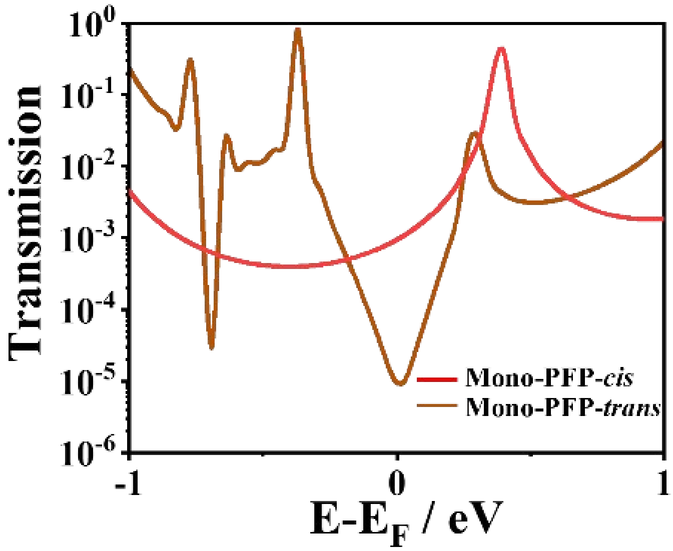

Fig. S12. (a) The *cis* isomer of **Mono-PFP**. (b) 1D conductance histograms of **Mono-PFP** under different biases. (c) 2D conductance histograms of **Mono-PFP** under different biases.

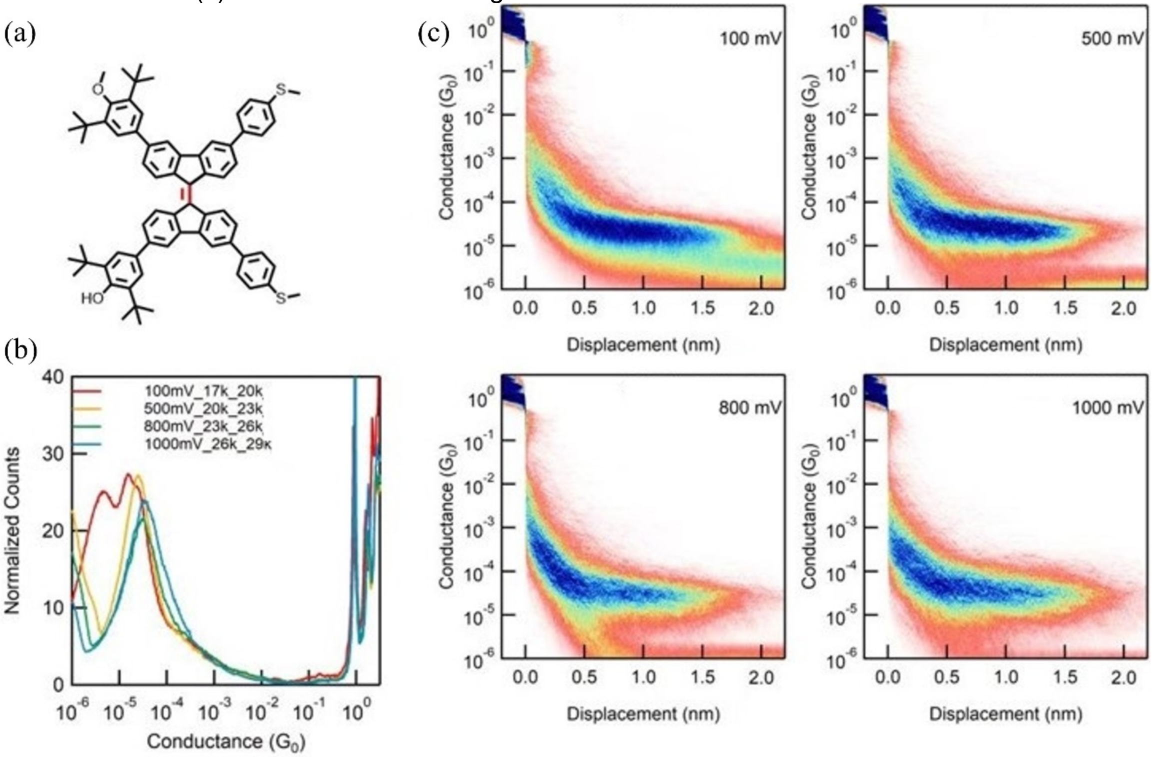

Fig. S13. 1D conductance histograms of three rounds of measurements on different batches of non-radicals.

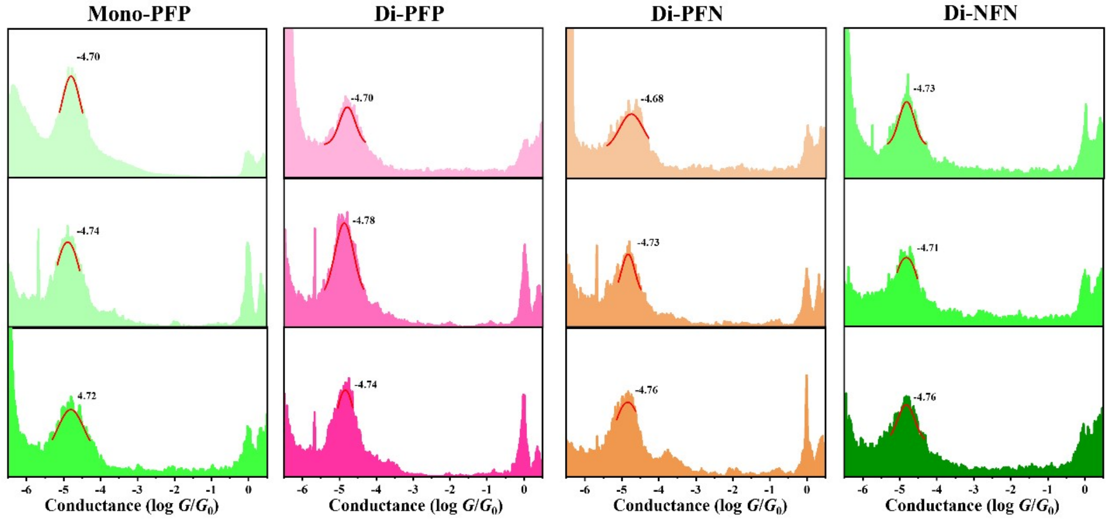

Fig. S14. 1D conductance histograms of three rounds of measurements on different batches of radicals.

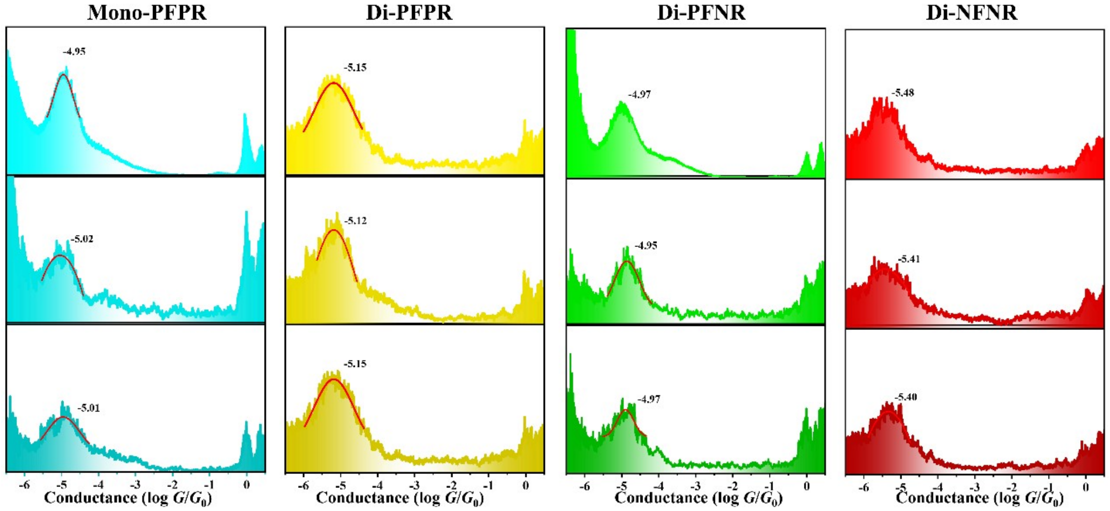

Fig. S15. The relative-displacement distributions of **Mono-PFPR**, **Di-PFPR**, **Di-PFNR** and **Di-NFNR**.

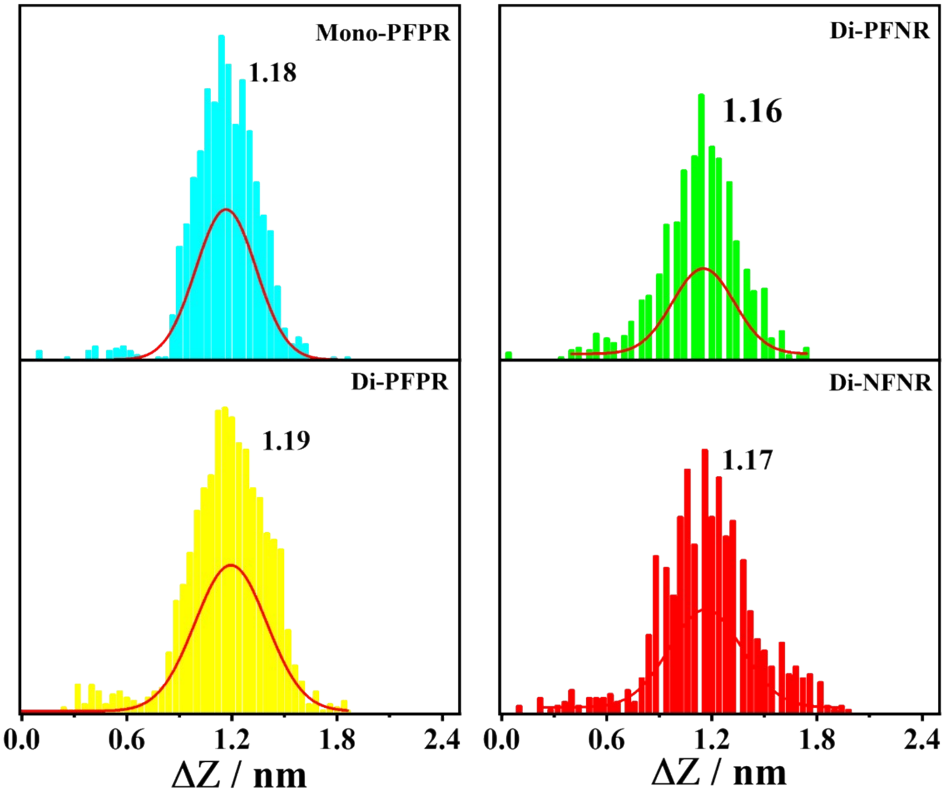

Fig. S16. 1D and 2D logarithmic conductance histograms of **4FR** and **4NR**.

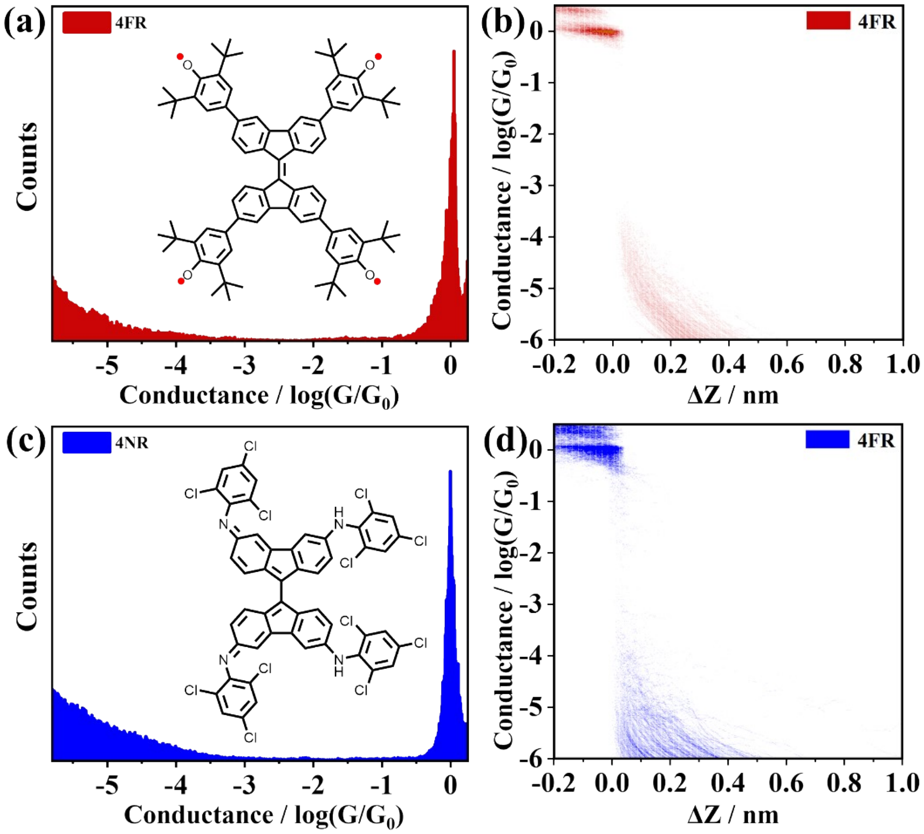

Fig. S17. 2D histogram of normalized conductance changes versus normalized noise power for **Mono-PFP**, **Di-PFP**, **Di-PFN**, **Di-NFNR**, **Mono-PFPR**, **Di-PFPR**, **Di-PFNR** and **Di-NFNR**.

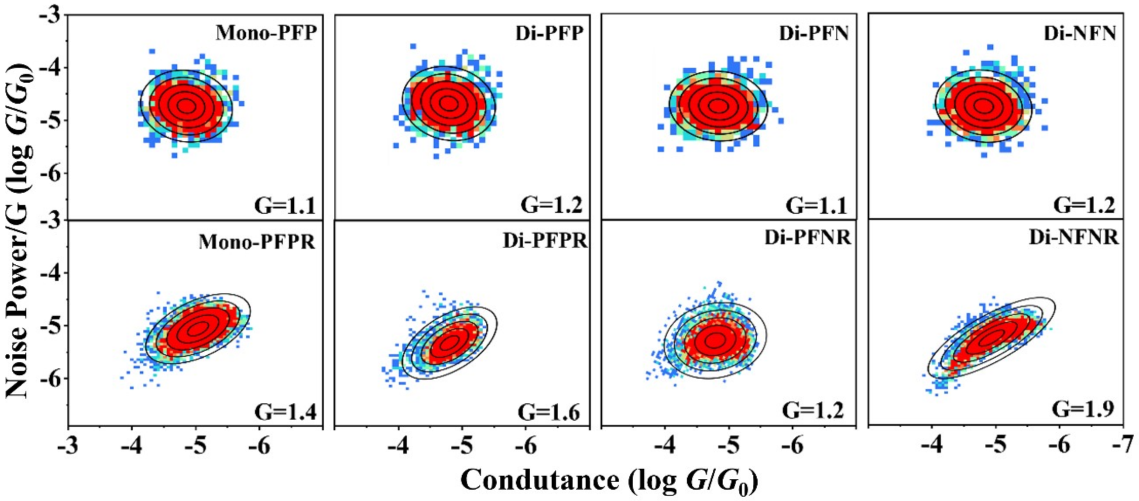

Fig. S18. The theoretical calculation of the torsion angle *θ* and C=C double bonds for **Mono-PFP**, **Mono-PFPR**, **Di-PFPR**, **Di-PFNR** and **Di-PFNR**.

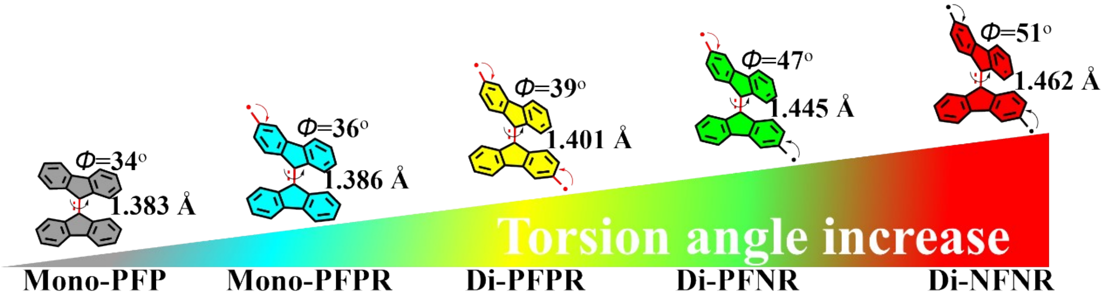

Fig. S19. Theoretical transport properties. Transmission spectra of plotted semi-logarithmically vs. energy relative to the Fermi energy for **Mono-PFP**, **Di-PFP**, **Di-PFN** and **Di-NFN**.

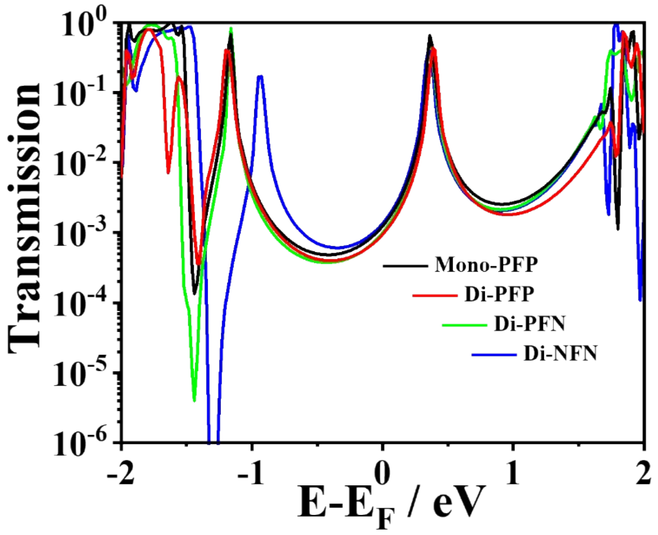

SI References

1. Tang, C. et al. Multicenter-Bond-Based Quantum Interference in Charge Transport Through Single-Molecule Carborane Junctions. *Angew. Chem. Int. Ed.* **58**, 10601-10605 (2019).
2. Liu, J. et al. Transition from Tunneling Leakage Current to Molecular Tunneling in Single-Molecule Junctions. *Chem* **5**, 390-401 (2019).
3. Wu, C. et al. Folding a Single-Molecule Junction. *Nano Lett.* **20**, 7980-7986 (2020).
4. Tang, C. et al. Reversible Switching between Destructive and Constructive Quantum Interference Using Atomically Precise Chemical Gating of Single-Molecule Junctions. *J. Am. Chem. Soc.* **143**, 9385-9392 (2021).
5. Lu, T. & Chen, F. Multiwfn: A multifunctional Wavefunction Analyzer. *J. Comput. Chem.* **33**, 580-592 (2012).
6. Humphrey, W., Dalke, A. & Schulten, K. VMD: Visual Molecular Dynamics. *J. Mol. Graph. Model.* **14**, 33-38 (1996).

NMR Spectra

^{1}H-NMR of **T1** in $CDCl_3$.

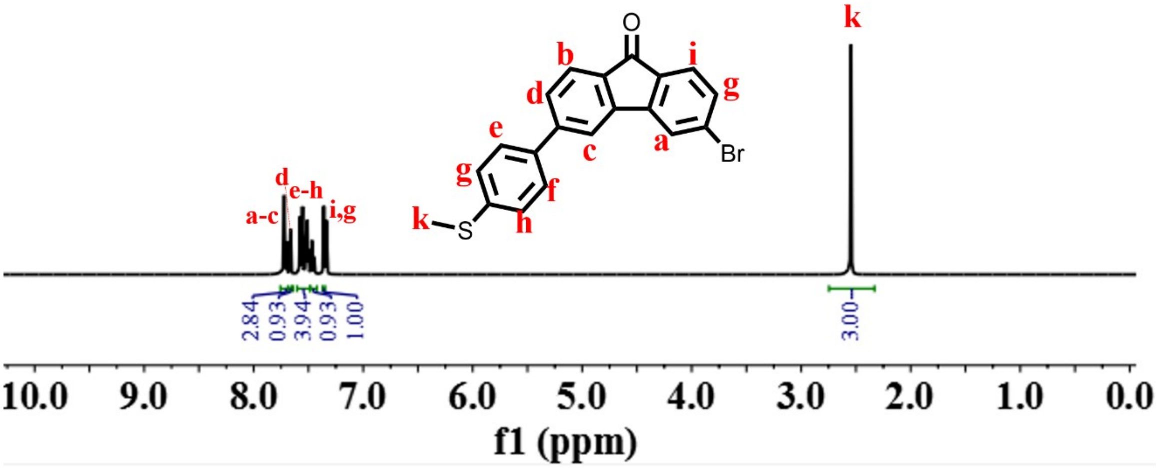

^{13}C-NMR of **T1** in $CDCl_3$.

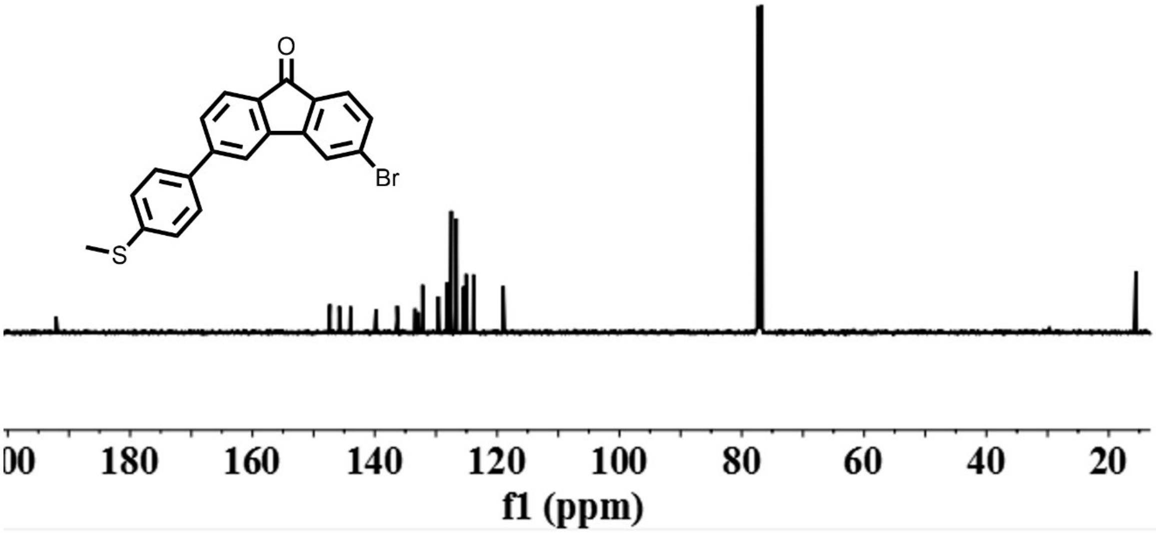

^{1}H-NMR of **T2** in $CDCl_3$.

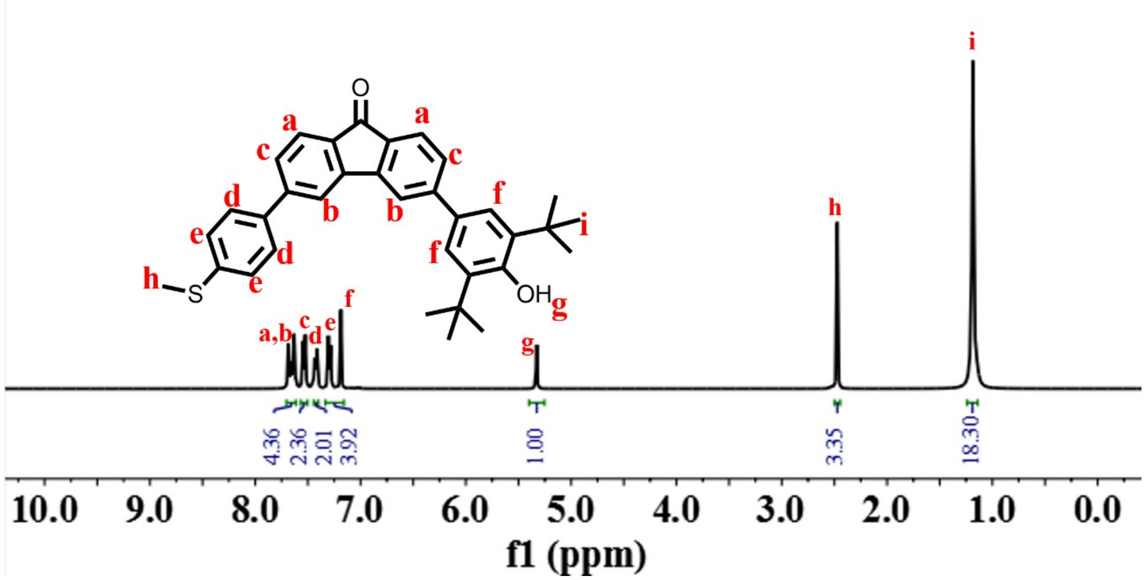

^{13}C-NMR of **T2** in $CDCl_3$.

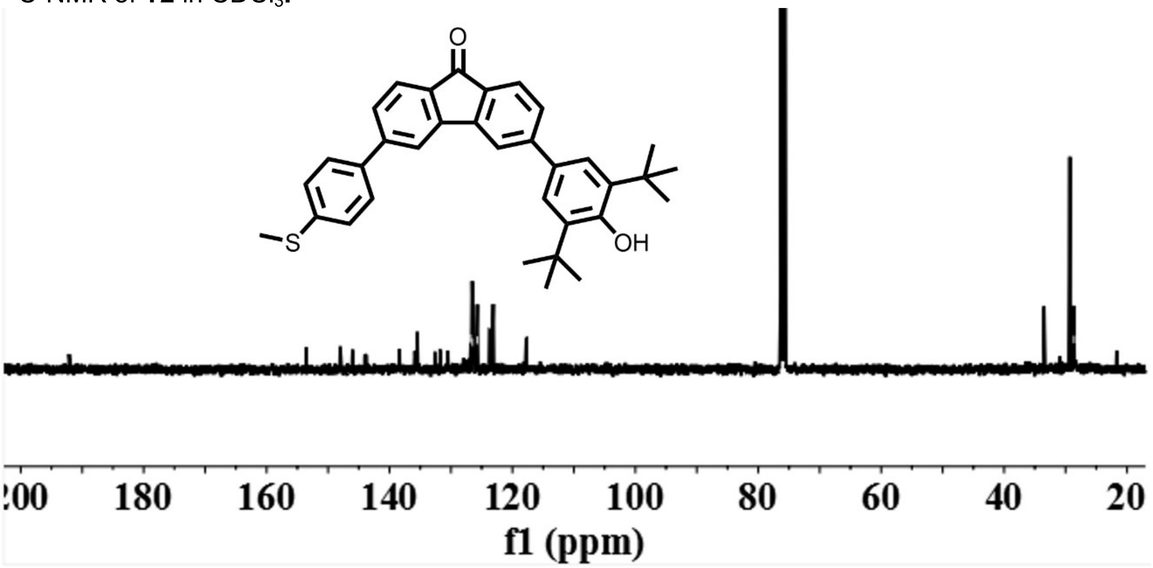

^{1}H-NMR of **T3** in $CDCl_3$.

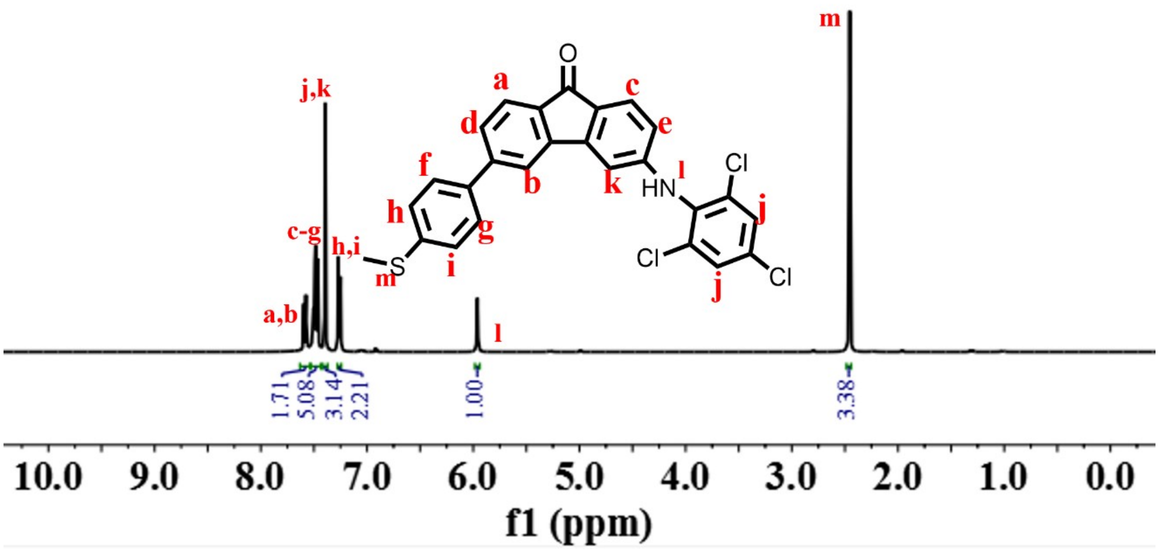

^{13}C-NMR of **T3** in $CDCl_3$.

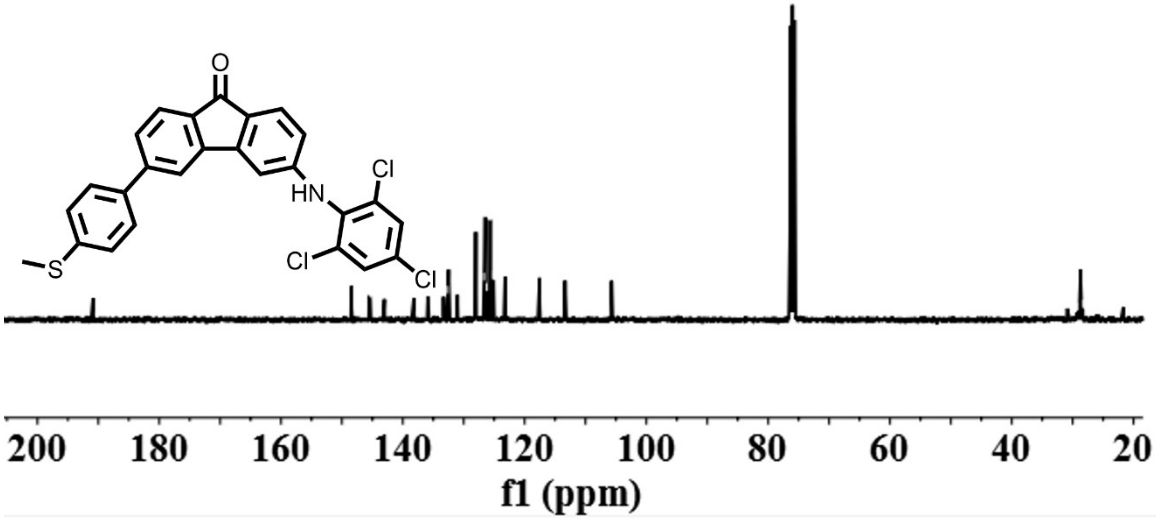

^{1}H-NMR of **Di-PFP** in $CDCl_3$.

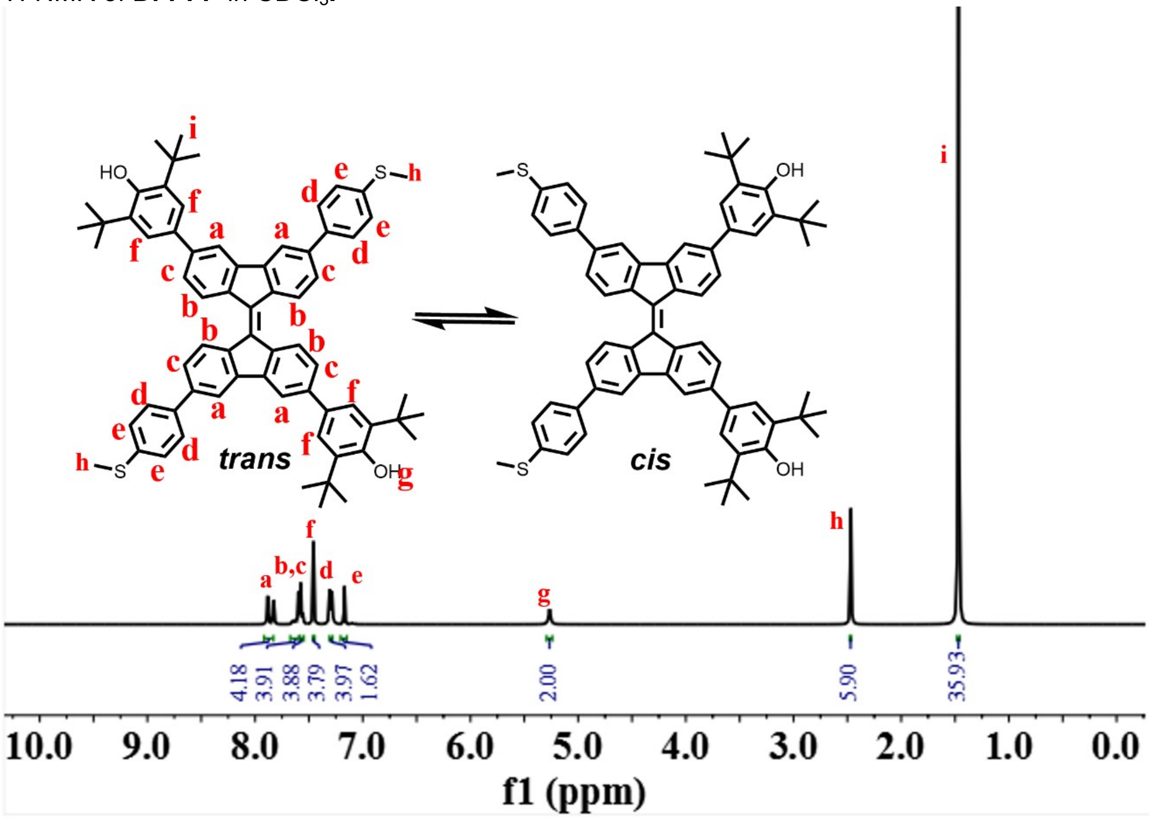

^{13}C-NMR of **Di-PFP** in $CDCl_3$.

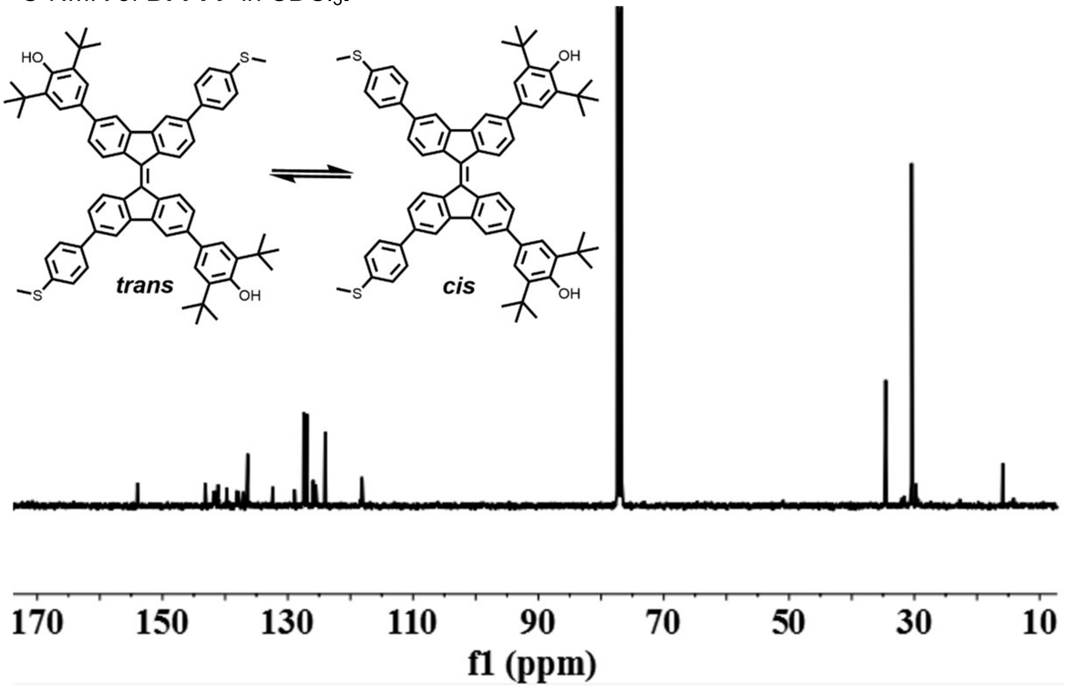

^{1}H-NMR of **Di-PFN** in $CDCl_3$.

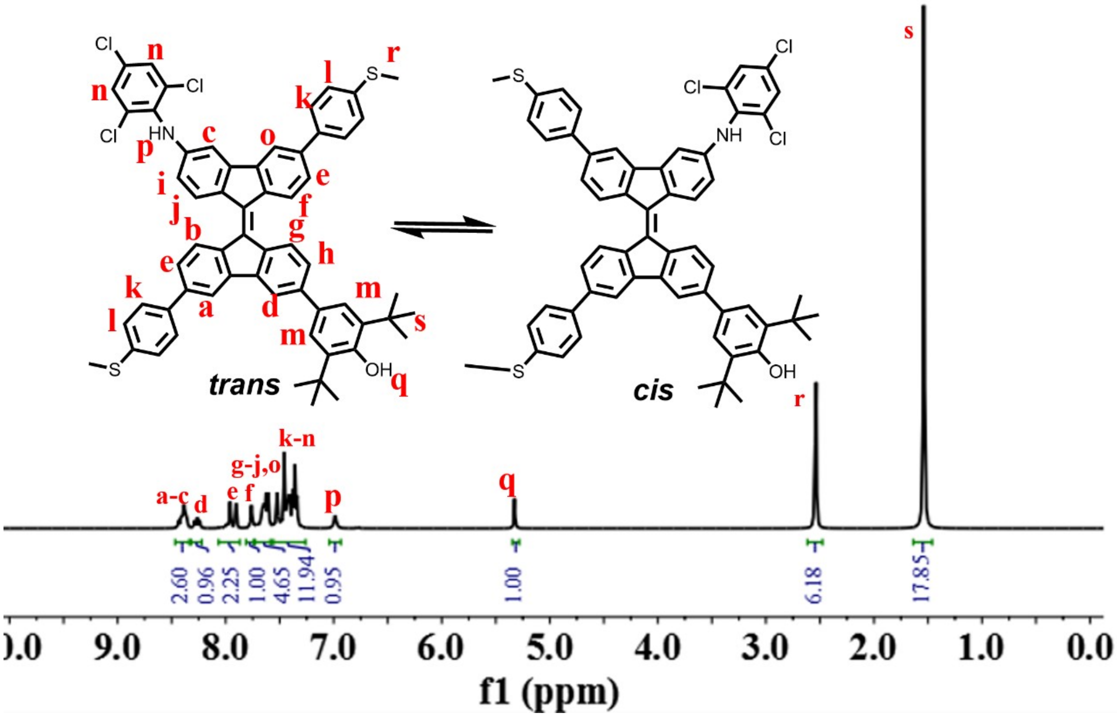

^{13}C-NMR of **Di-PFN** in $CDCl_3$.

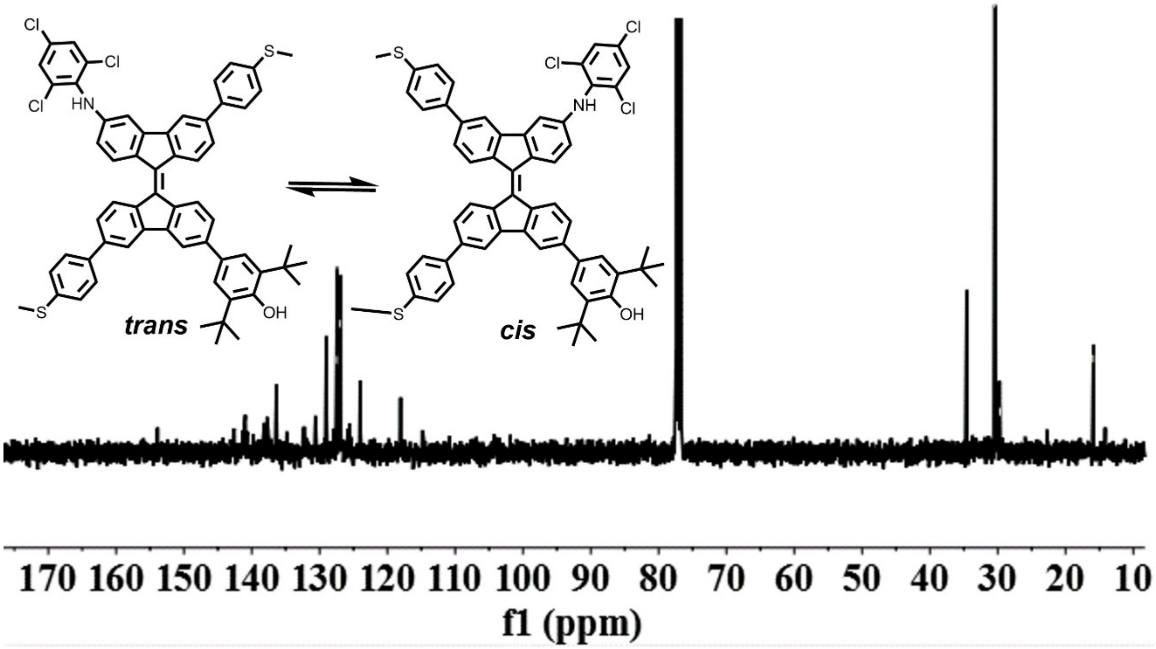

^{1}H-NMR of **Di-NFN** in $CDCl_3$.

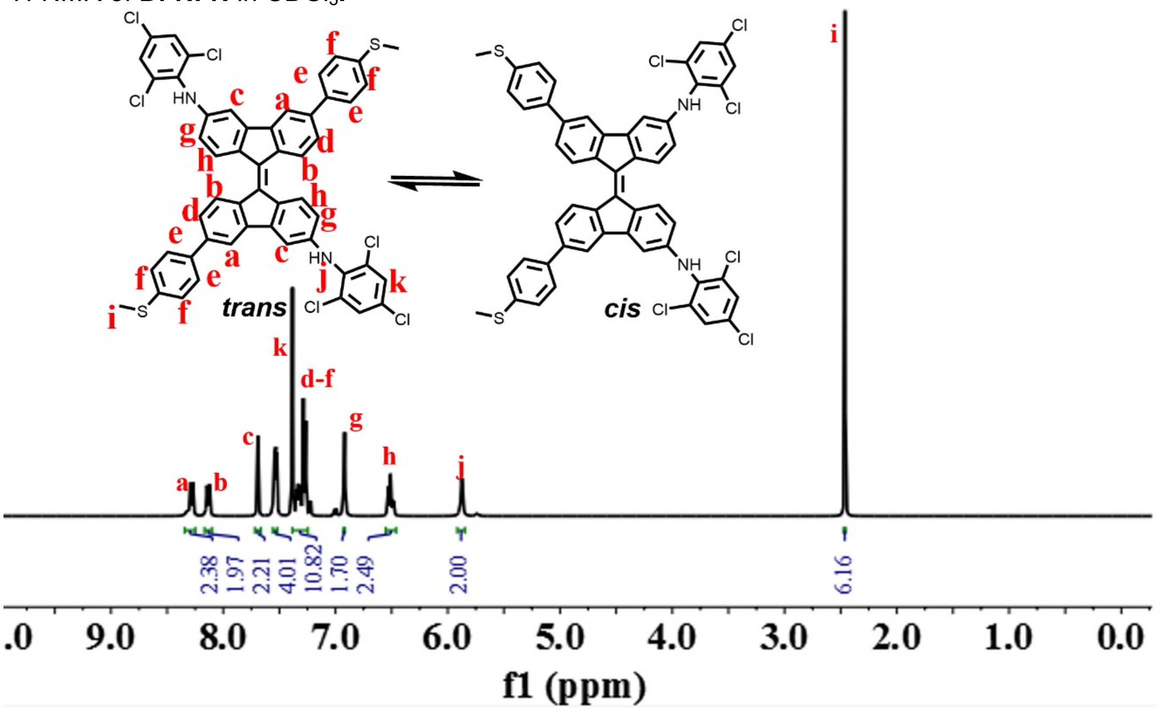

^{13}C-NMR of **Di-NFN** in $CDCl_3$.

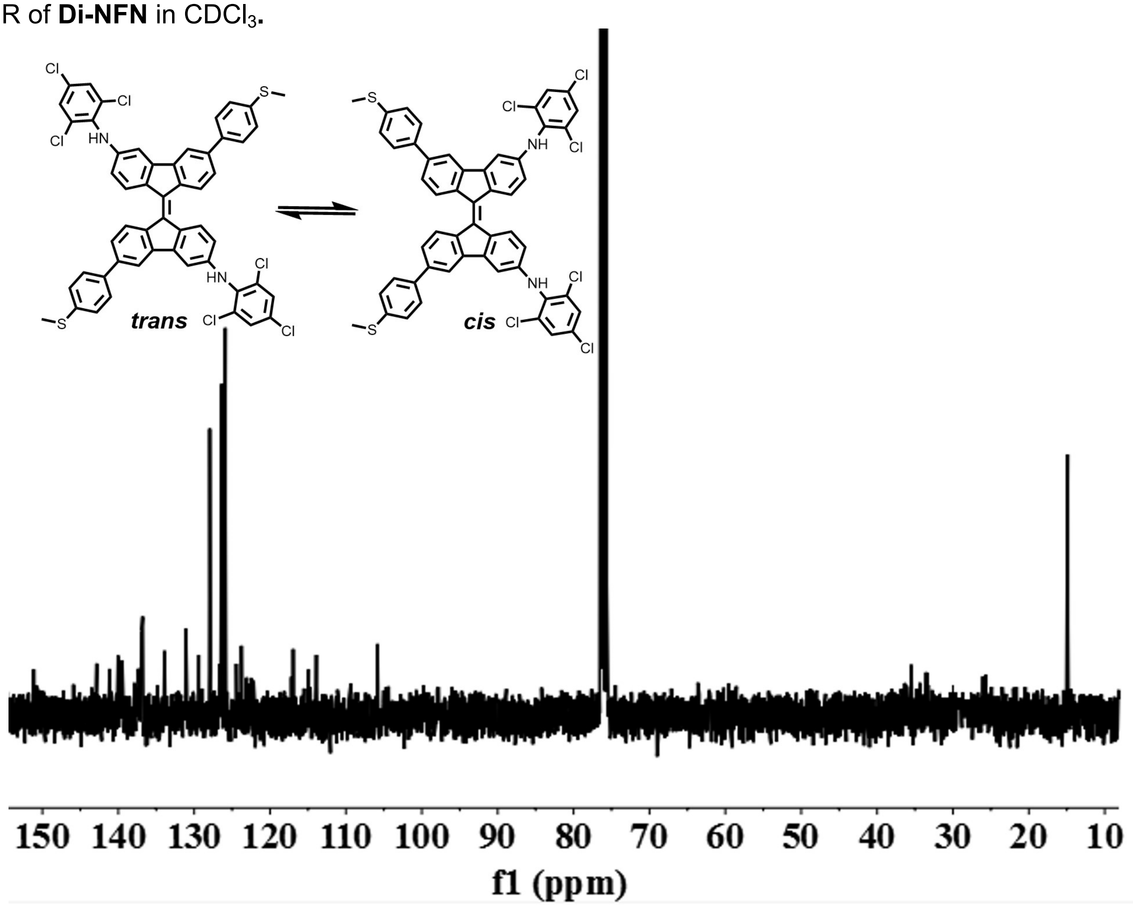

^{1}H-NMR of **Mono-PFP** in $CDCl_3$.

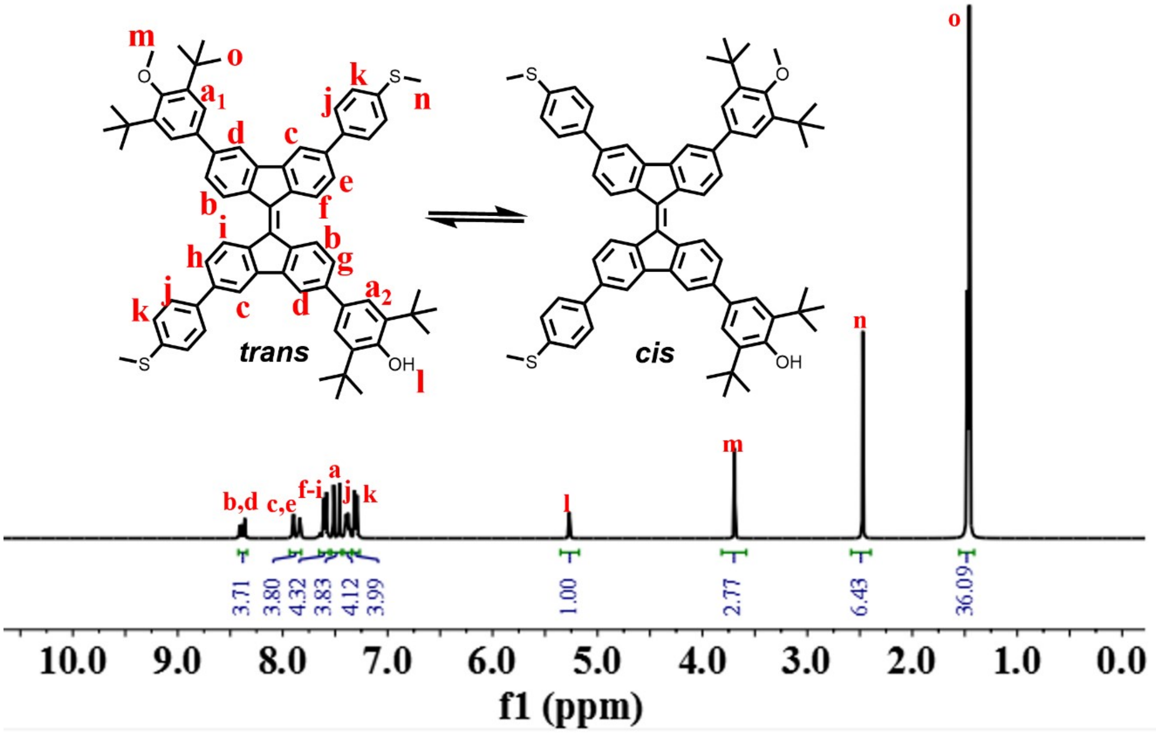

^{13}C-NMR of **Mono-PFP** in $CDCl_3$.

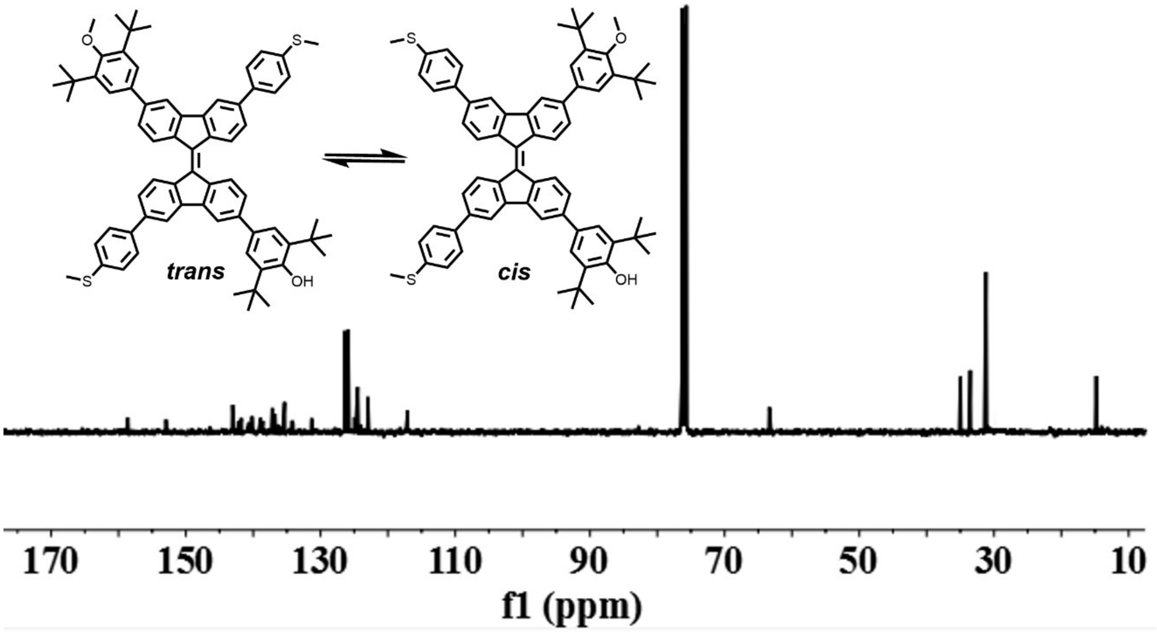

^{1}H-NMR of **Di-NFNR** in $CDCl_3$.

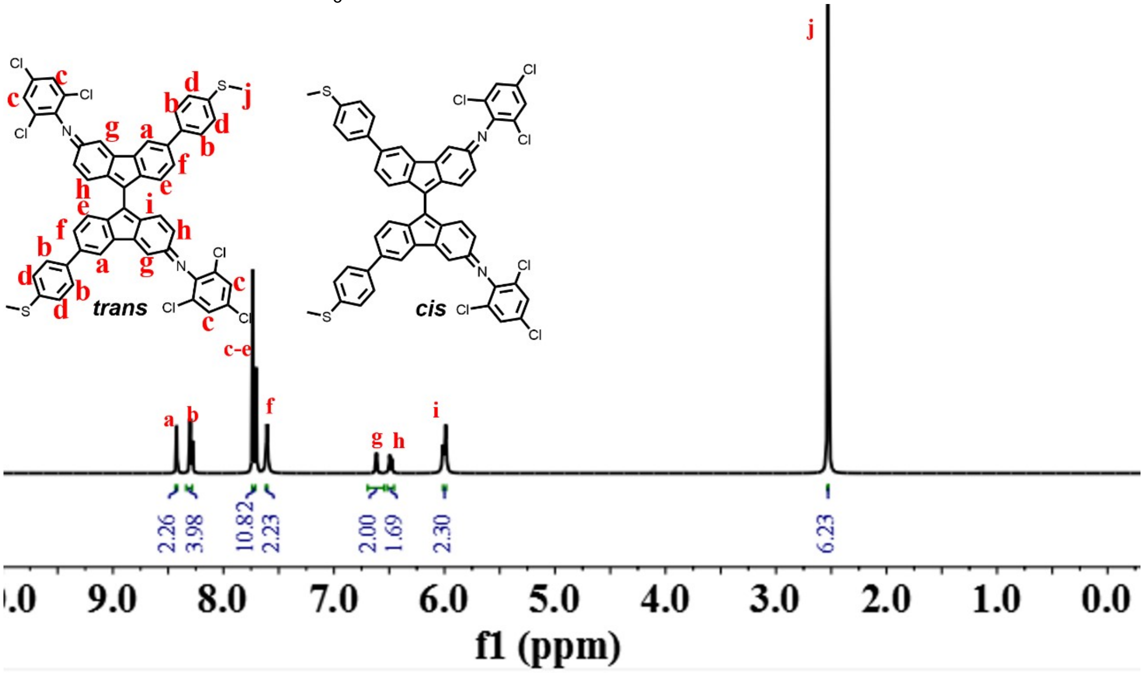

Mass Spectra

Maldi-Tof mass spectra of **T1**

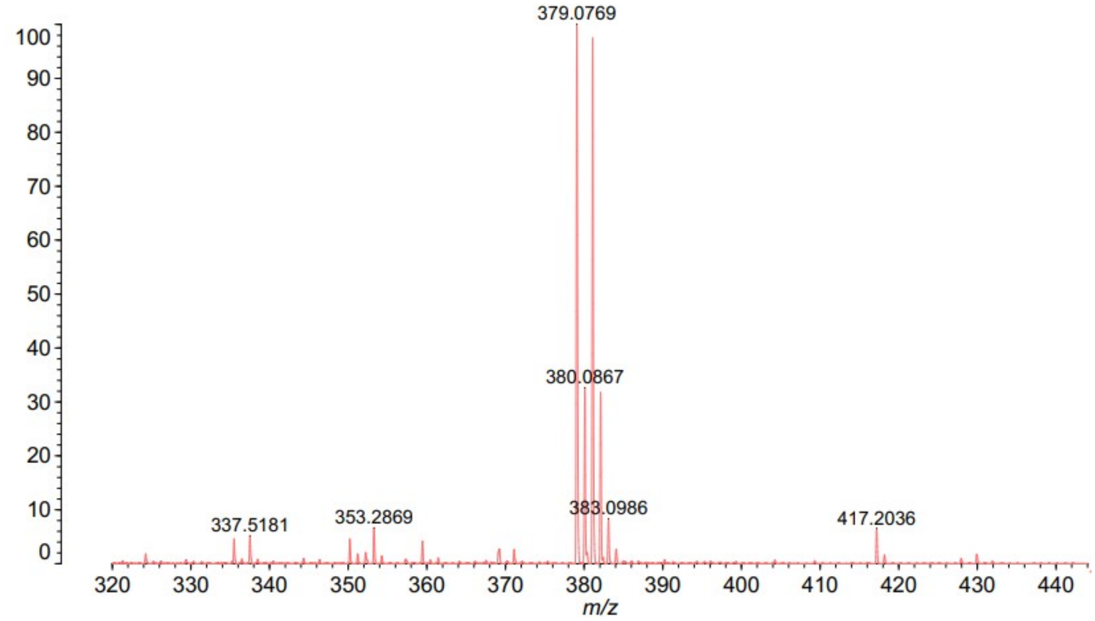

Maldi-Tof mass spectra of **T2**

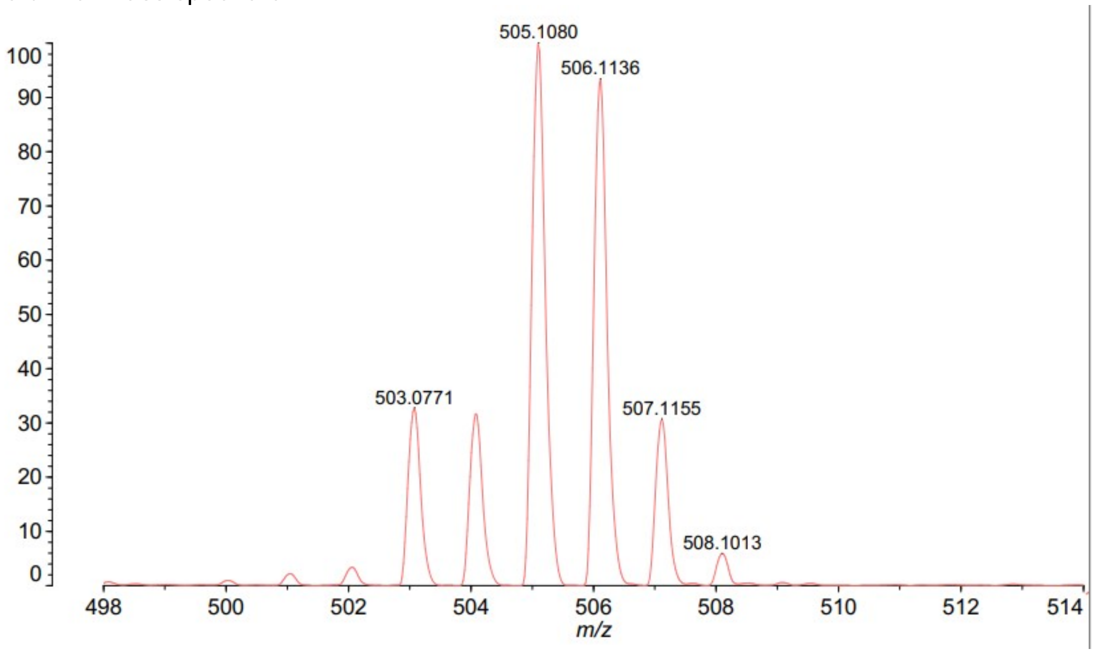

Maldi-Tof mass spectra of **T3**

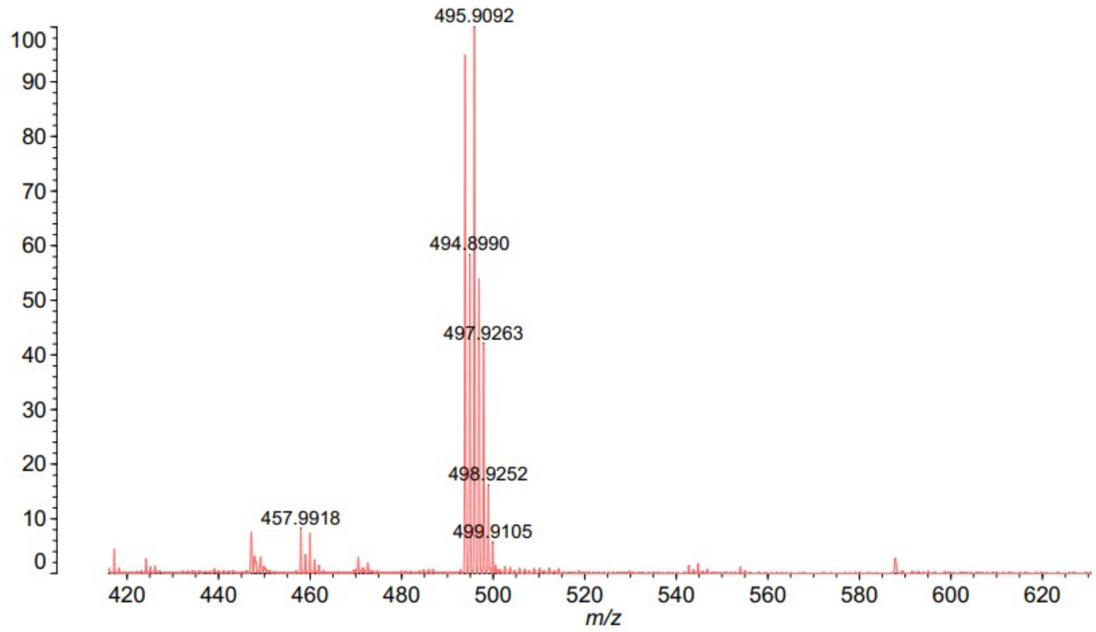

Maldi-Tof mass spectra of **Di-PFP**

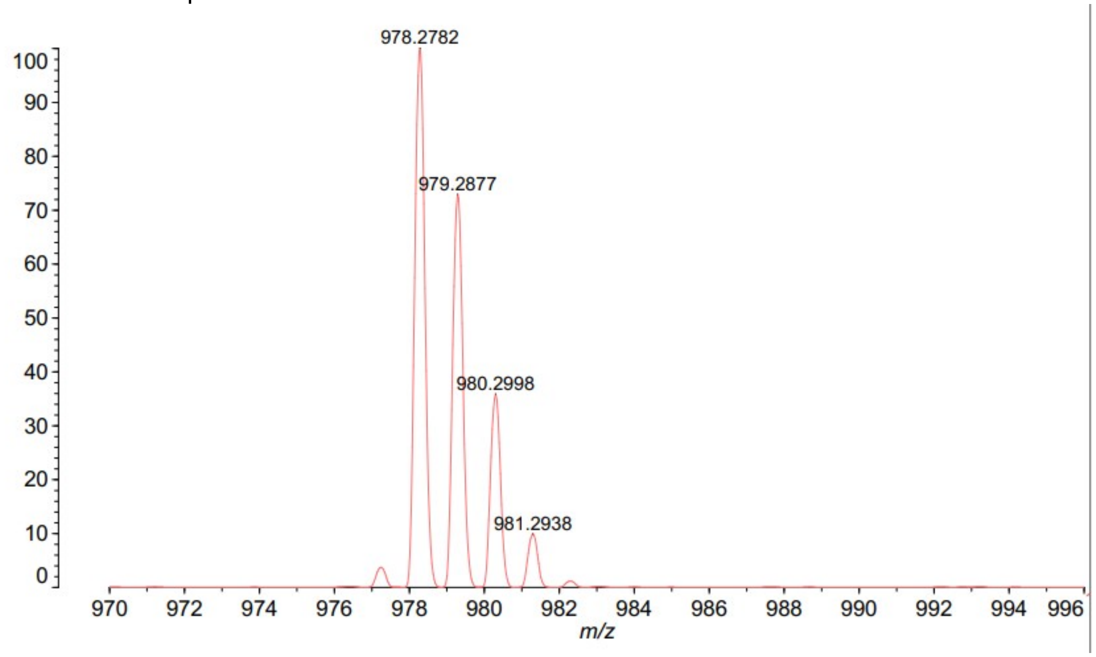

Maldi-Tof mass spectra of **Di-PFPR**

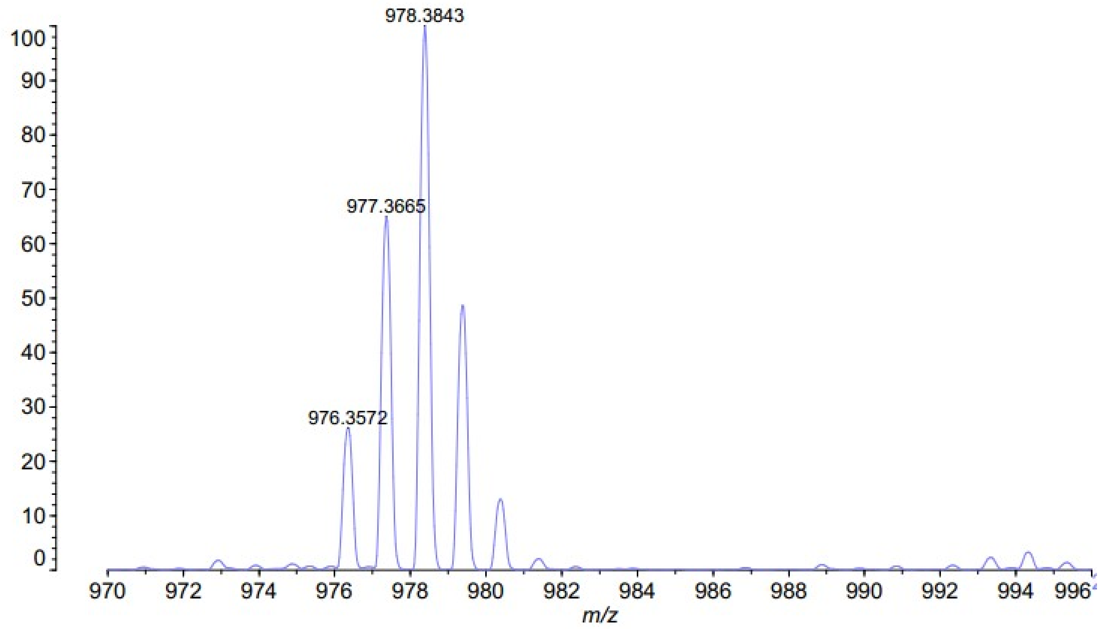

Maldi-Tof mass spectra of **Di-PFN**

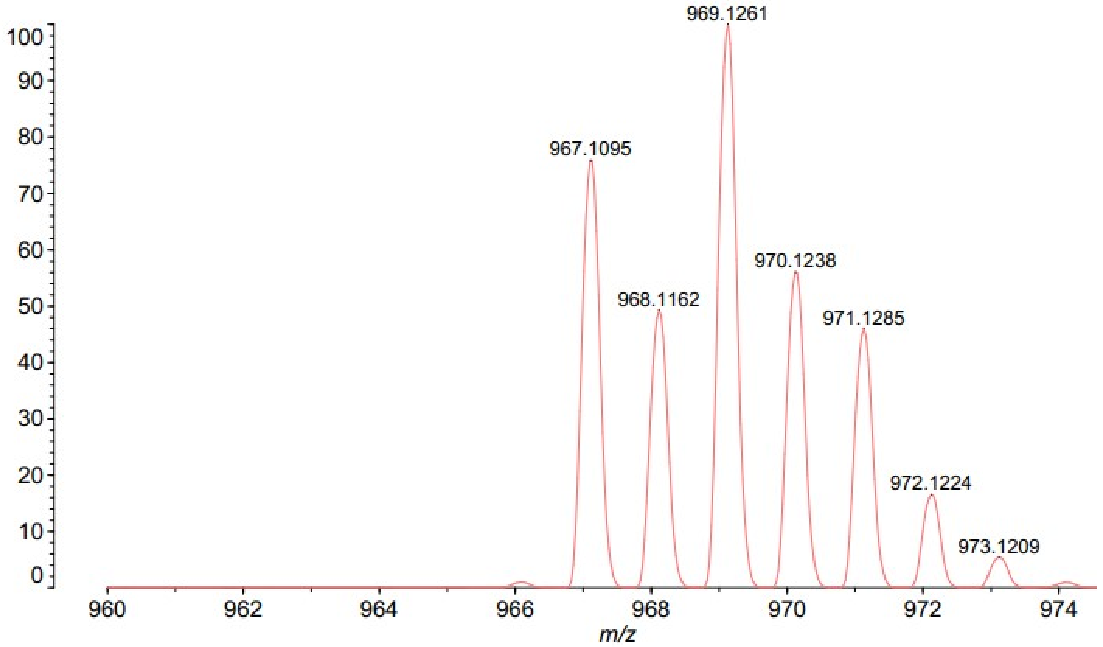

Maldi-Tof mass spectra of **Di-PFNR**

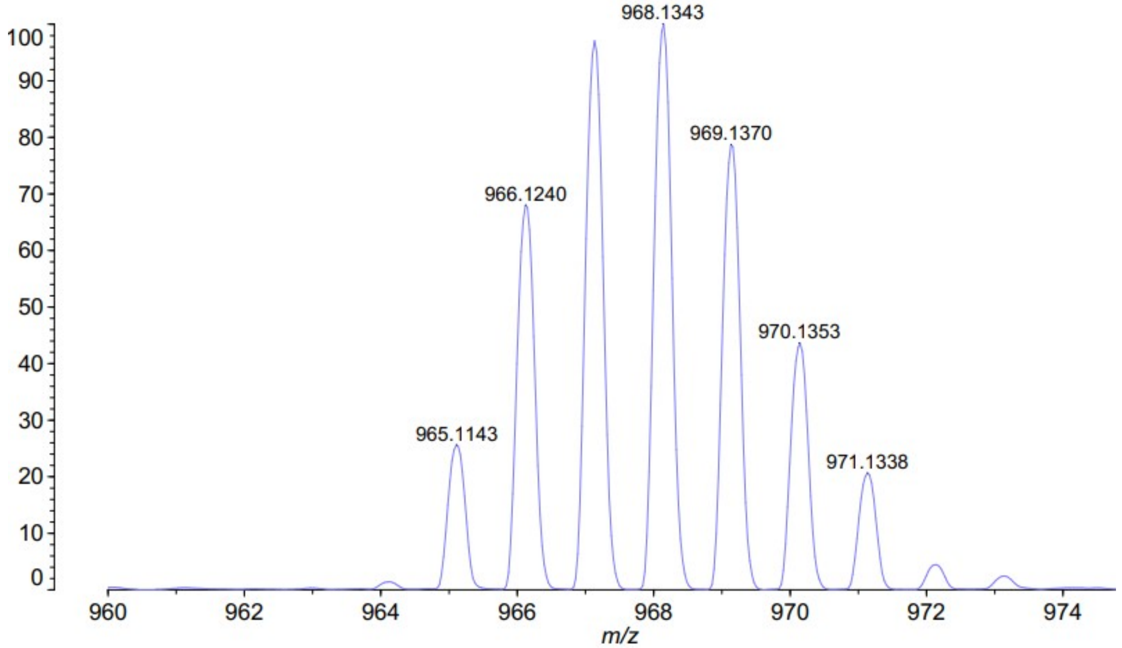

Maldi-Tof mass spectra of **Di-NFN**

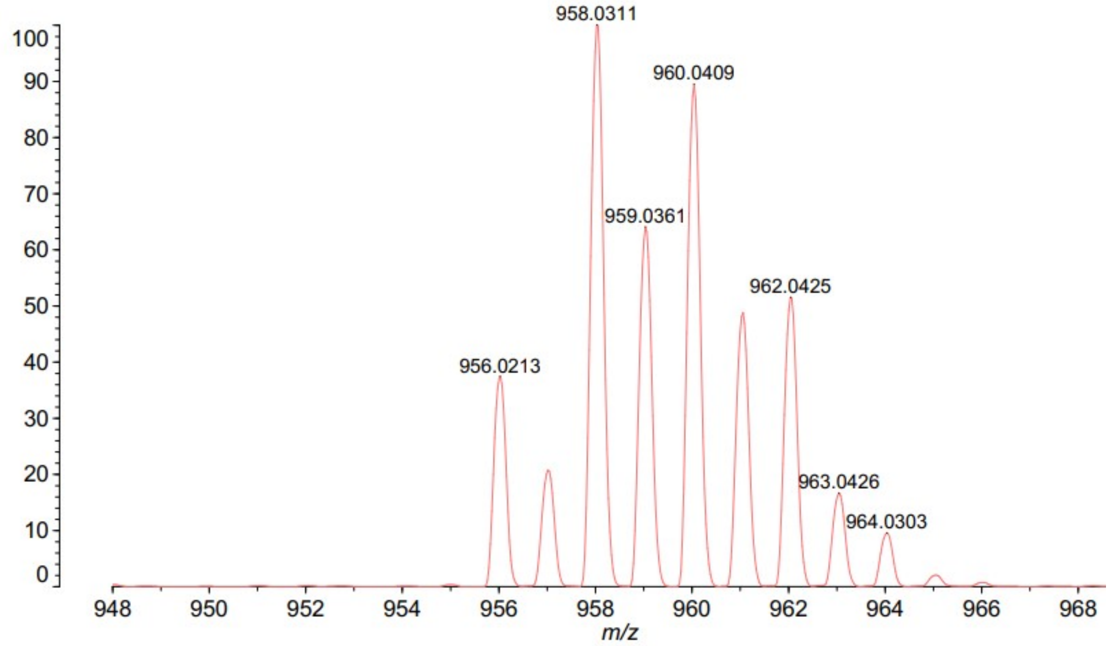

Maldi-Tof mass spectra of **Di-NFNR**

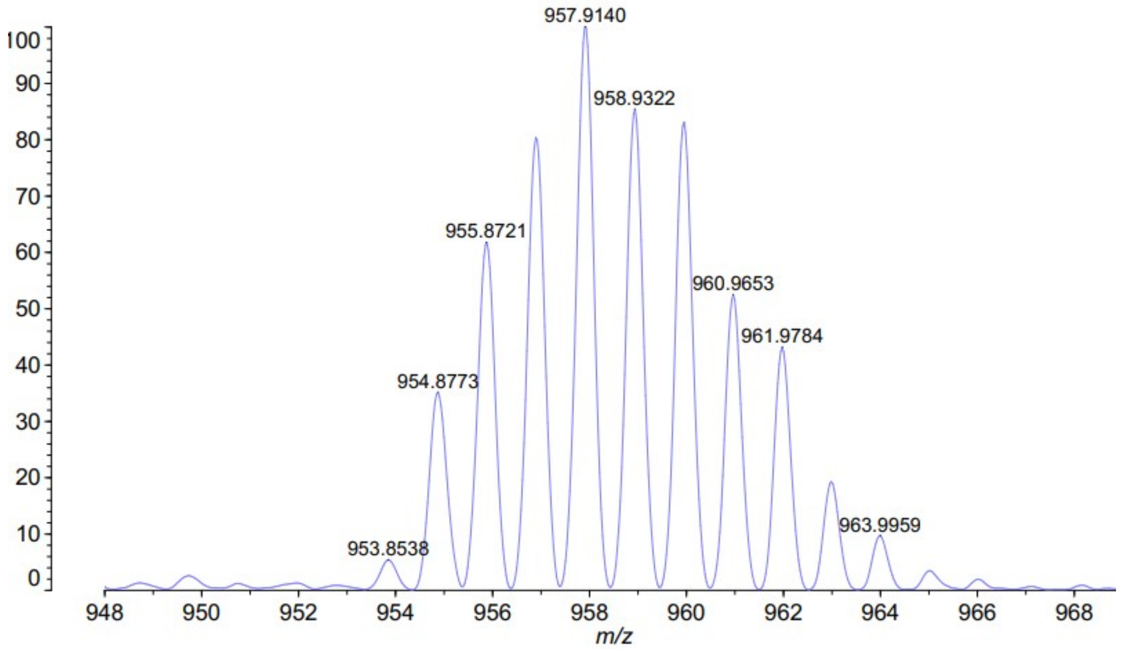

Maldi-Tof mass spectra of **Mono-PFP**

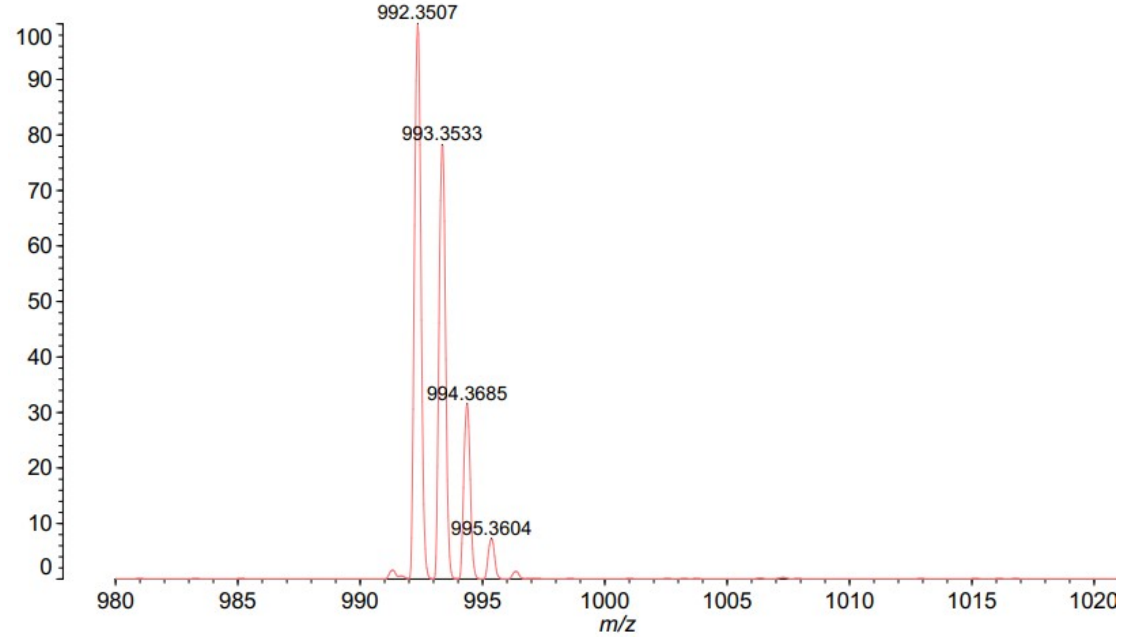

Maldi-Tof mass spectra of **Mono-PFPR**

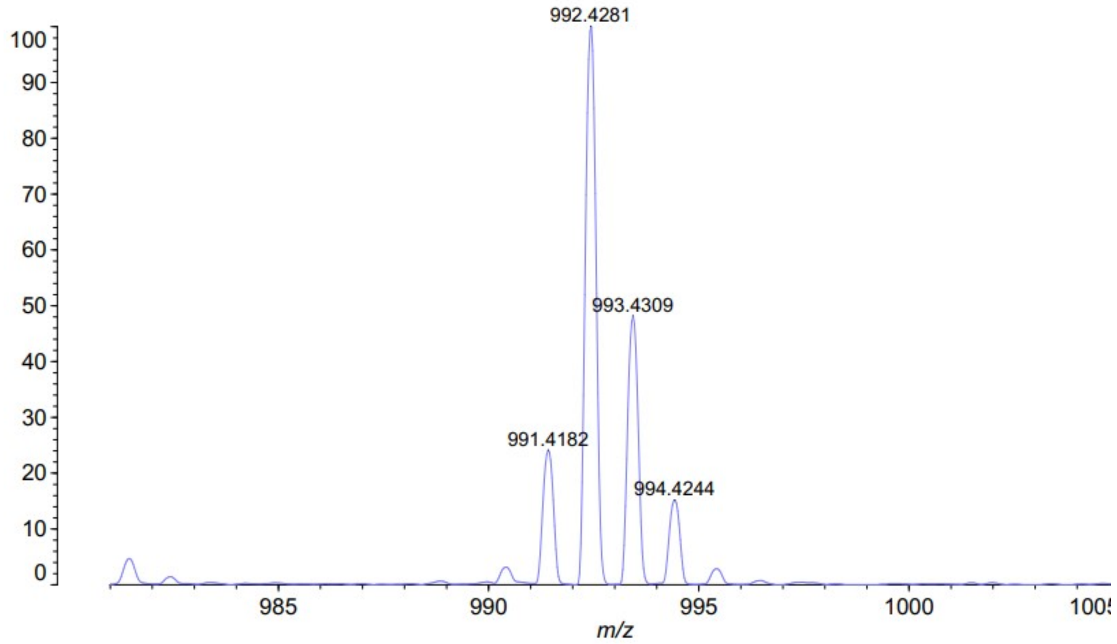

Maldi-Tof mass spectra of **4FR**

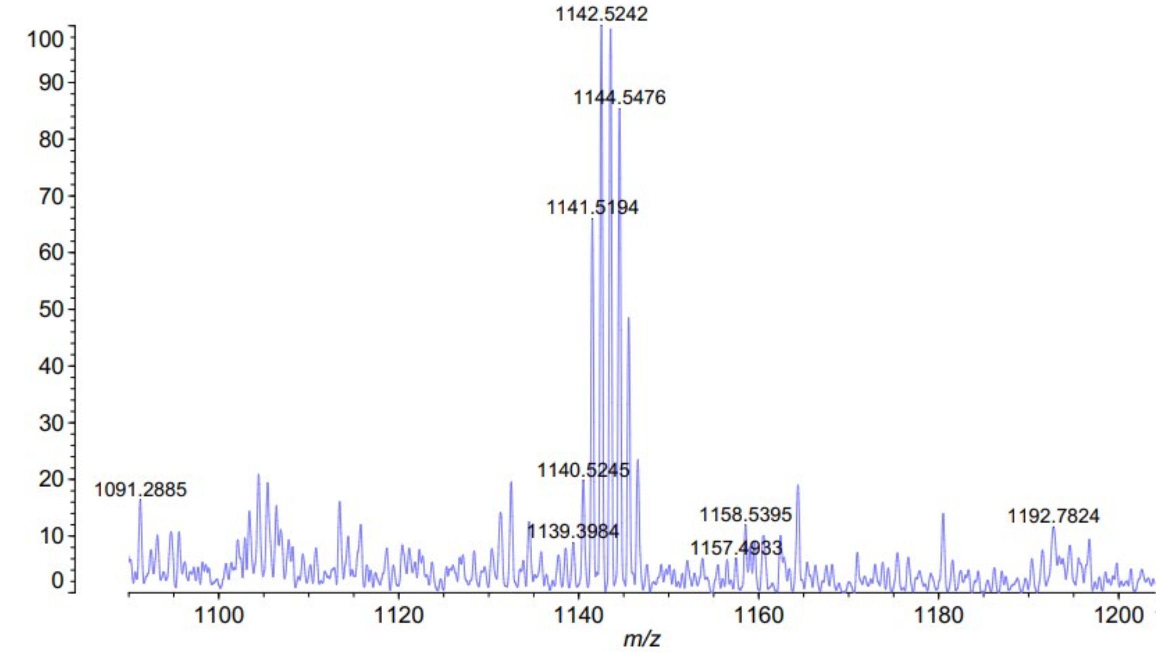

Maldi-Tof mass spectra of **4NR**

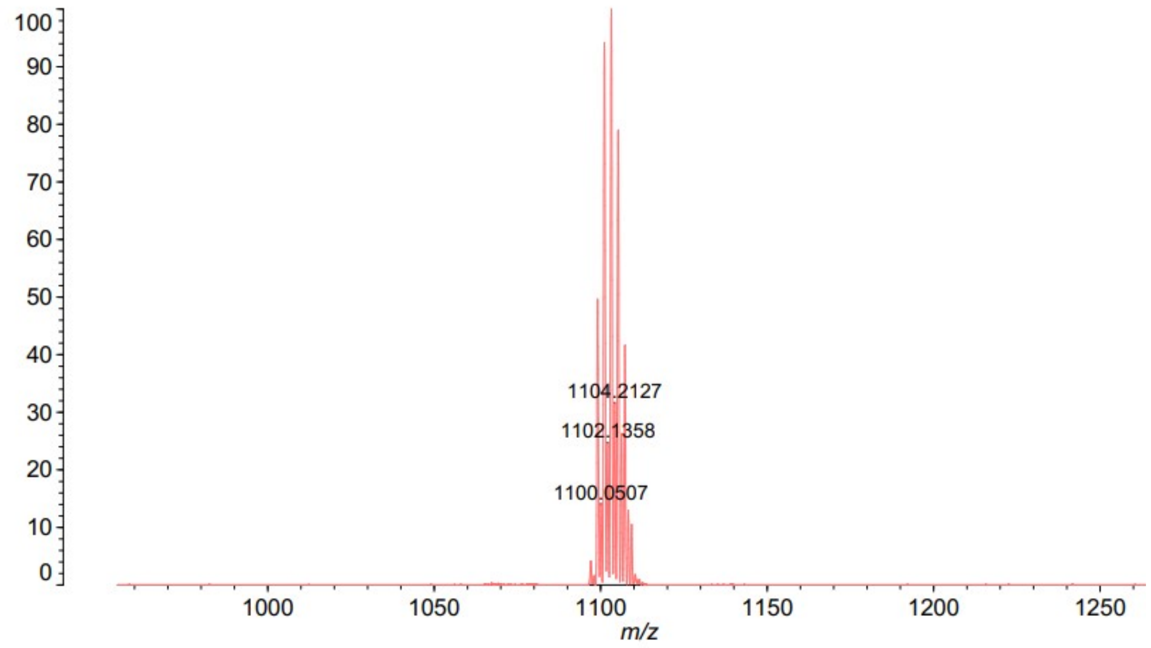

HRMS spectra

HRMS spectra of **Mono-PFPR**

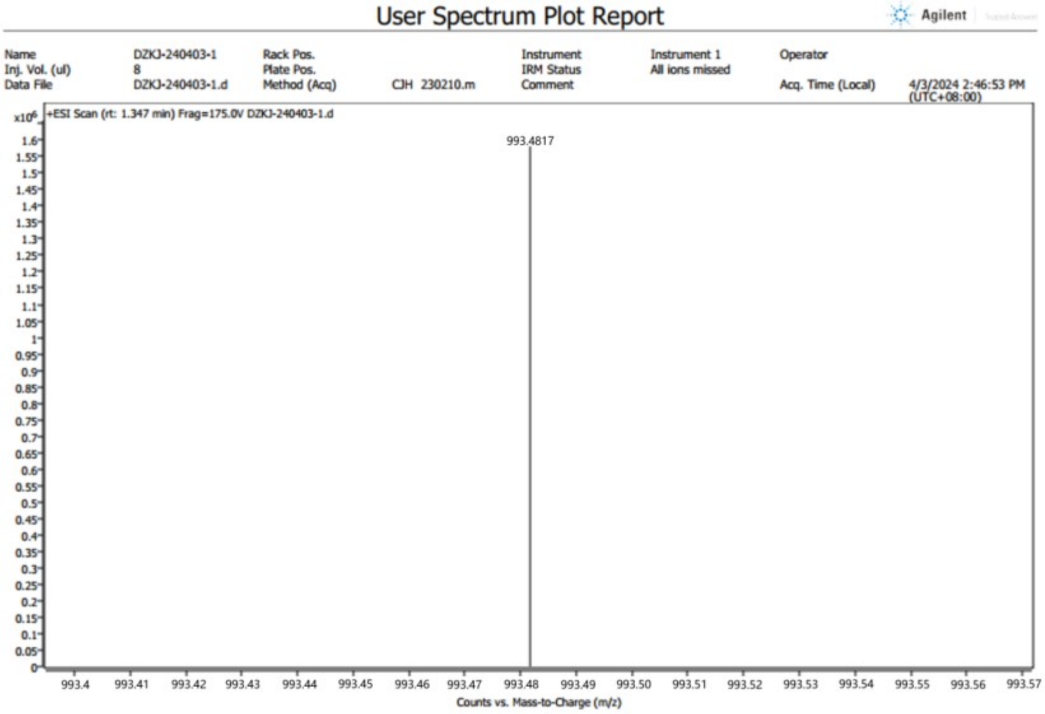

HRMS spectra of **Di-PFPR**

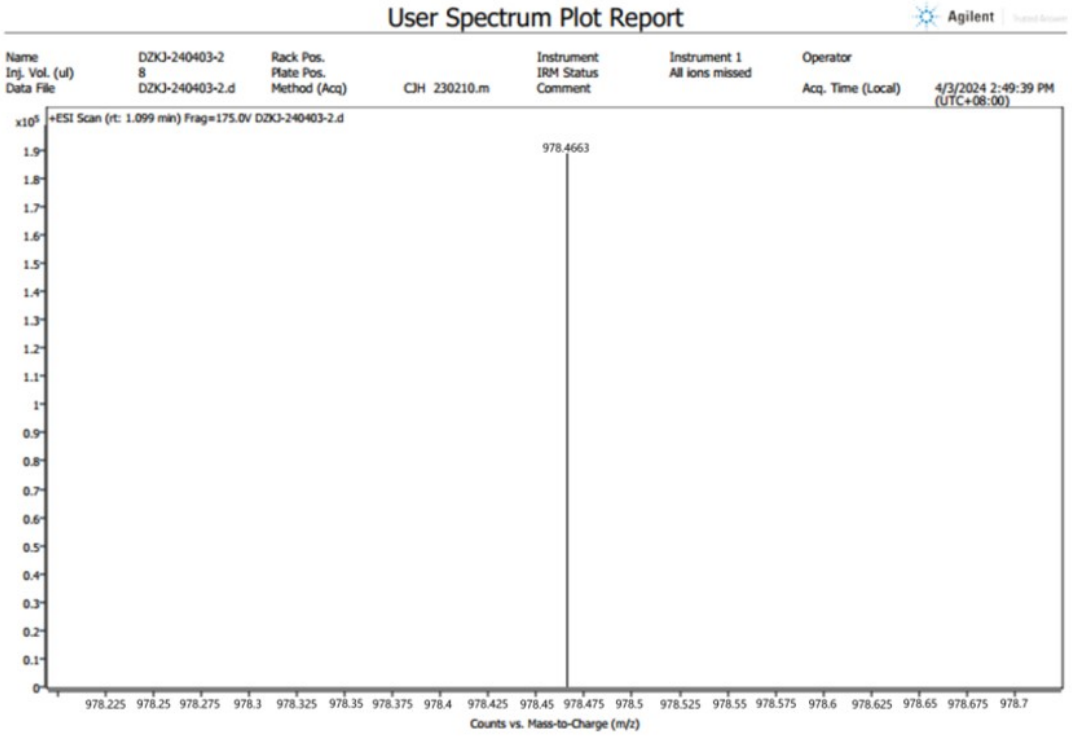

HRMS spectra of **Di-PFNR**

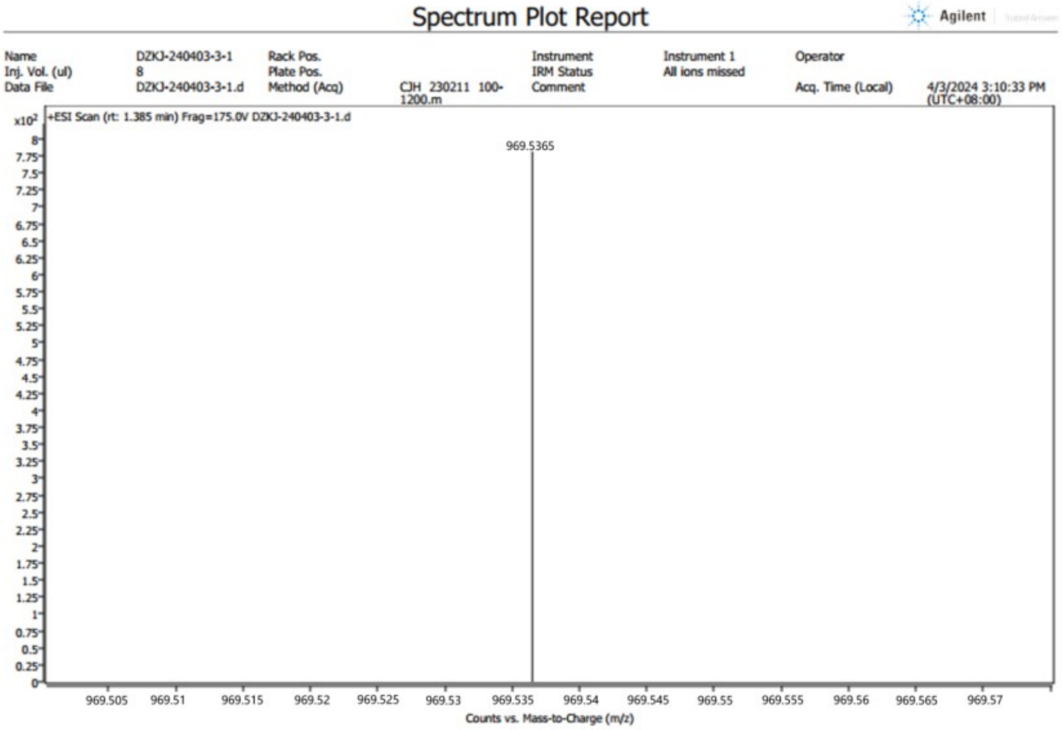

HRMS spectra of **Di-NFNR**

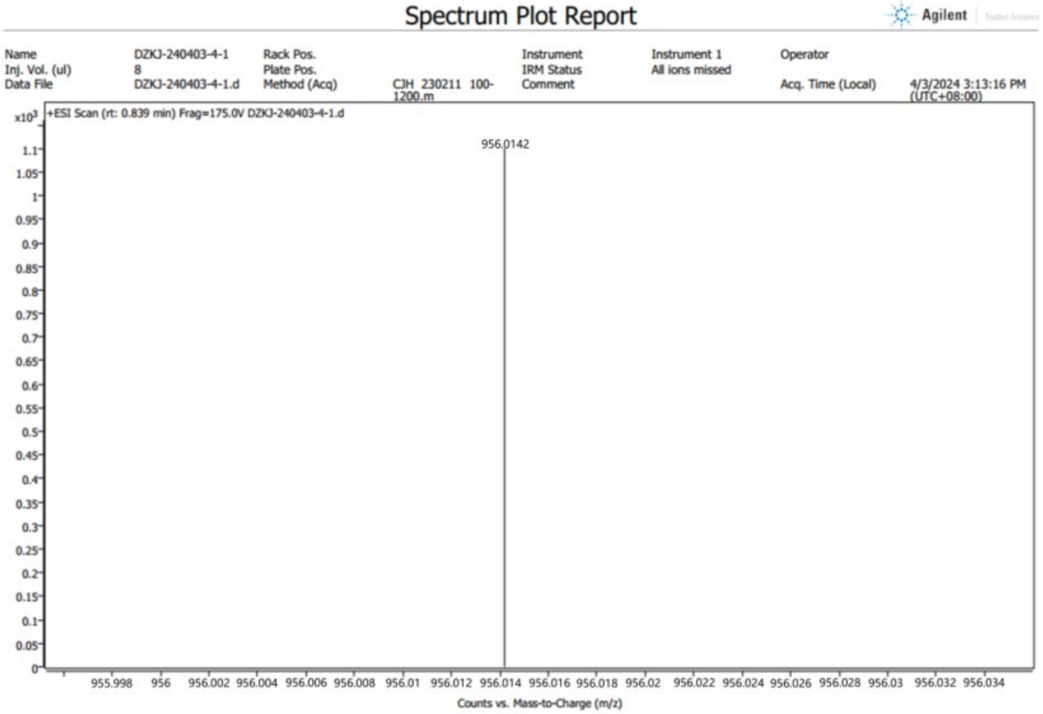

Supplement: SC-016-D4SC07256A-s001 [file SC-016-D4SC07256A-s001.pdf]
